# *Solenostomus snuffleupagus* sp. nov., a hairy ghost pipefish (Teleostei: Solenostomidae) from the Southwest Pacific, with an integrative comparison to *S. paegnius*

**DOI:** 10.1111/jfb.70497

**Published:** 2026-05-10

**Authors:** Graham Short, David Harasti

**Affiliations:** ^1^ Australian Museum Research Institute Sydney New South Wales Australia; ^2^ California Academy of Sciences San Francisco California USA; ^3^ Burke Museum of Natural History and Culture Seattle Washington USA; ^4^ University of Technology Sydney Sydney New South Wales Australia; ^5^ Port Stephens Fisheries Institute Taylors Beach New South Wales Australia

**Keywords:** coral reefs, micro‐CT, mitochondrial DNA, new species, Syngnathiformes, taxonomy

## Abstract

A new species of ghost pipefish, *Solenostomus snuffleupagus* sp. nov., is described from the Coral Sea based on specimens (18–34 mm SL) collected from coral reef habitats in Queensland, Australia. The species is diagnosed by the following combination of characters: abundant elongate integumentary filaments imparting a conspicuously shaggy appearance; 36 total vertebrae (vs. 32–34 in all congeners); compact body form with a short, deep pretrunk (11%–14% SL); sexually dimorphic supraoccipital crests (moderately elevated in females, strongly developed in males); and two modified anchor‐like ossicles spanning the pterygiophores in both soft dorsal and anal fins. Micro‐CT imaging reveals distinctive pretrunk ossicle configuration characterized by deep angular interspaces and absence of a ventral ossicle series. Mitochondrial COI sequences indicate an uncorrected p‐distance of 22.0% from the superficially similar *S. paegnius*, a species with which it has long been confused in museum collections and citizen science databases. Field observations document consistent association with dense filamentous red macroalgae, with body coloration closely matching host substrate. The species is currently known from the southwest Pacific, including northeastern Australia (Coral Sea), Papua New Guinea, New Caledonia, Fiji and Tonga, representing a southwestern Pacific distribution.

## 1 INTRODUCTION

The ghost pipefishes (Solenostomidae Lacépède 1803) are a small family of cryptic syngnathiform fishes phylogenetically sister to Syngnathidae, which includes seahorses, pipefishes and sea dragons (Dawson, 1985; Hamilton et al., 2017; Stiller et al., 2022). These solenostomids possess an elongated tubular snout and small terminal mouth, but differ fundamentally from syngnathids in their reproductive biology, in which females, rather than males, brood eggs within a marsupium formed by the united pelvic fins (Jungersen, 1910; Orr & Fritzsche, 1993).

Ghost pipefish are distributed throughout the tropical Indo‐Pacific, from the Red Sea and East Africa eastward to Australia, the central Pacific and Japan (Kuiter, 2009; Orr & Fritzsche, 1993). They inhabit diverse environments ranging from coral reefs to sandy and muddy substrates, seagrass meadows and algal beds, at depths from the surface to at least 95 m. All species exhibit remarkable crypsis, frequently mimicking algae, seagrasses, crinoids and soft corals in both form and coloration. Their appendages and pigmentation patterns provide effective camouflage among benthic substrates, with some species closely resembling filamentous algae or, in the case of *S. halimeda*, the calcareous green macroalga *Halimeda* (Orr et al., 2002).

There are currently six recognized species within *Solenostomus*: *S. cyanopterus* (Bleeker, 1854), widespread from the Red Sea to the western Pacific; *S. paradoxus* (Pallas, 1770), ranging from the Indian Ocean to the western Pacific; *S. paegnius* (Jordan & Thompson, 1914), distributed throughout the Indo–West Pacific; *S. leptosoma* (Tanaka, 1908), occurring in the western Pacific including Japan, Taiwan, Indonesia and the Philippines; *S. halimeda* (Orr et al., 2002), associated with *Halimeda* algae in northwest Australia, Indonesia, Papua New Guinea and the Philippines; and *S. armatus*.

Weber (1913), redescribed by Matsuura (2017) from southern Japan. Despite this modest diversity, taxonomic resolution within the genus has been hindered by extreme crypsis, rarity of specimens in museum collections and significant intraspecific variation in meristic and morphometric characters (Orr & Fritzsche, 1993). Filamentous forms within *Solenostomus* have proven particularly problematic, with continued uncertainty as to whether they represent distinct species or morphological variants of *S. cyanopterus* or *S. paegnius* (Araga, 1967). This taxonomic confusion has persisted in part because *S. paegnius* has remained poorly characterized in recent literature, with limited osteological data available for comparison. Resolving species boundaries among filamentous ghost pipefishes requires integrative analysis combining external morphology, skeletal characters and molecular data.

Underwater photography and citizen science observations have increasingly facilitated recognition of undescribed diversity within *Solenostomus*. A distinctive filamentous ghost pipefish with consistently orange to red coloration and a conspicuously hairy appearance has long been observed in the southwest Pacific, particularly on the Great Barrier Reef (GBR) near Cairns, Queensland, Australia and in Fiji, where it is well known to local divers. The earliest documented material consists of two specimens collected in 1993 in northern Queensland by the Australian Museum (AMS) and Museum and Art Gallery of the Northern Territory (NTM). Since 2005, the species has been regularly recorded at Saxon and Norman Reefs, GBR, largely through diver observations shared on citizen science platforms such as Facebook groups (e.g., GBR Macro Critters) and iNaturalist.

Owing to its superficial resemblance to *S. paegnius*, particularly in possessing elongate filamentous appendages, and the absence of formal taxonomic treatment, this species has remained unidentified in scientific records. It is frequently misidentified on citizen science platforms, with erroneous records also appearing from New Caledonia, Papua New Guinea and Fiji. Despite its effective camouflage among red macroalgae of similar form and hue, consistent morphological differences from *S. paegnius* and other congeners warrant recognition as a distinct species.

The present study formally describes this species as *S. snuffleupagus* sp. nov., based on integrative analysis of morphological, osteological and molecular data. Direct comparison with *S. paegnius* reveals diagnostic differences in body form, vertebral count, neurocranial morphology, pretrunk ossicle arrangement and genetic divergence. Furthermore, we extend the known range of *S. paegnius* from the western Pacific into the western Indian Ocean, documenting the first museum specimen from South Africa housed in the South African Institute for Aquatic Biodiversity (SAIAB), previously misidentified as *S. cyanopterus*.

This integrative analysis definitively separates *S. snuffleupagus* sp. nov. from *S. paegnius* and clarifies the diagnostic characters of both species. The integration of high‐resolution micro‐CT imaging with traditional morphological and molecular approaches provides new insights into the osteological diversity of *Solenostomus* and demonstrates the value of citizen science observations in documenting cryptic marine biodiversity.

## 2 MATERIALS AND METHODS

### 2.1 Material examined

All specimens of *Solenostomus snuffleupagus* sp. nov. and comparative material of *S. paegnius* (Jordan & Thompson, 1914) are listed in the Material Examined section following the Discussion. Comparative specimens of *S. paegnius* were selected to encompass the known geographic range and morphological variation of that species, allowing for a robust morphometric and osteological comparison with the new species.

### 2.2 Terminology

Throughout this study, we use the term ossicles to describe the discrete, star‐shaped bony elements that comprise the dermal skeletal structure in *Solenostomus*. These stellate ossicles are embedded within the soft integument and arranged in transverse and longitudinal series across the body surface. Their shape, arrangement and ornamentation contribute significantly to the external morphology of the genus. In previous studies, these elements have been referred to as scutes (Jungersen, 1910) and plates (Orr & Fritzsche, 1993); however, we adopt the term ossicles to more accurately reflect their discrete, non‐overlapping and variably branched forms.

The regional classification of ossicle series follows a modified version of traditional plate‐ and scute‐based terminology. Pretrunk ossicles are those located anterior to the first (spinous) dorsal fin. Trunk ossicles lie posterior to the first dorsal fin and anterior to the origins of the soft dorsal and anal fins, including those forming the last complete ossicular ring. Tail ossicles extend from the bases of the soft dorsal and anal fins to the caudal‐fin base, excluding the terminal median ossicle. The posterior portion of this series, positioned behind the complete ossicular ring at the level of the soft dorsal and anal fin insertions, is referred to as caudal peduncle ossicles. Modified ossicles at the bases of the soft dorsal and anal fins are identified separately as dorsal‐fin ossicles and anal‐fin ossicles, respectively. Neurocranial and postcranial skeletal terminology follows Jungersen (1910) and Short and Trnski (2021). Head and body measurements follow Short et al. (2018) and are expressed as percentages of standard length (SL) or head length (HL).

### 2.3 Measurements

Morphometric and meristic data were obtained from high‐resolution digital images and measured to the nearest 0.1 mm using ImageJ v1.53 (Schneider et al., 2012). Measurements include SL, from the tip of the snout to the base of the caudal fin; HL, from the tip of the snout to the posterior margin of the opercle; pretrunk length (PreTrl), from the posterior edge of the opercle to the fifth pretrunk ossicular ring; trunk length (TrL), from the fifth pretrunk ossicular ring to the base of the caudal fin; caudal peduncle length (CPL), from the complete ossicular ring at the anal‐fin insertion to the caudal‐fin base; pretrunk depth (PreTrlD), measured vertically between the dorsalmost and ventralmost ossicles of the fifth pretrunk ossicular ring; snout depth (least and greatest, SnD), from the mandibular articulation to the anterior margin of the nasal capsule; and snout length (SnL), from the tip of the premaxilla to the anterior margin of the orbit. All proportional measurements are expressed as percentages of SL. Mean values and proportional measurements are presented in parentheses following observed ranges. Vertebral and fin ray counts were derived from CT imagery.

### 2.4 Specimen examination

Museum acronyms follow Sabaj (2020). Specimens were examined using a dissecting microscope and documented with high‐resolution digital photography (Canon EOS 5D Mark II with Canon EF 50 mm f/2.5 Compact Macro lens). Internal morphological characters were assessed through non‐destructive X‐ray micro‐computed tomography (μCT). Type specimens of *S. snuffleupagus* sp. nov. were scanned at the Australian Museum using a Nikon X‐Tek XT H 225 micro‐CT scanner at a voxel resolution of 8–15 μm, operating at 100–120 kV and 100–160 μA with a 0.25 mm copper filter. Scans were performed with moist specimens sealed in plastic and reconstructed using Skyscanner 3D. SUITE software. Comparative specimens of *S. paegnius* were scanned at the Karel F. Liem Bioimaging Facility (Friday Harbour Laboratories, University of Washington) using a Bruker Skyscan 1273 scanner (Bruker MicroCT, Billerica, MA, USA), equipped with a 1 mm aluminium filter, operating at 60 kV and 110 μA, with a 2048 × 2048 pixel CCD at a resolution of 4.5 μm. Volume rendering and segmentation of CT datasets were conducted using 3D Slicer v5.6 (Fedorov et al., 2012), an open‐source medical imaging platform.

### 2.5 Molecular analyses

**FIGURE 1:**
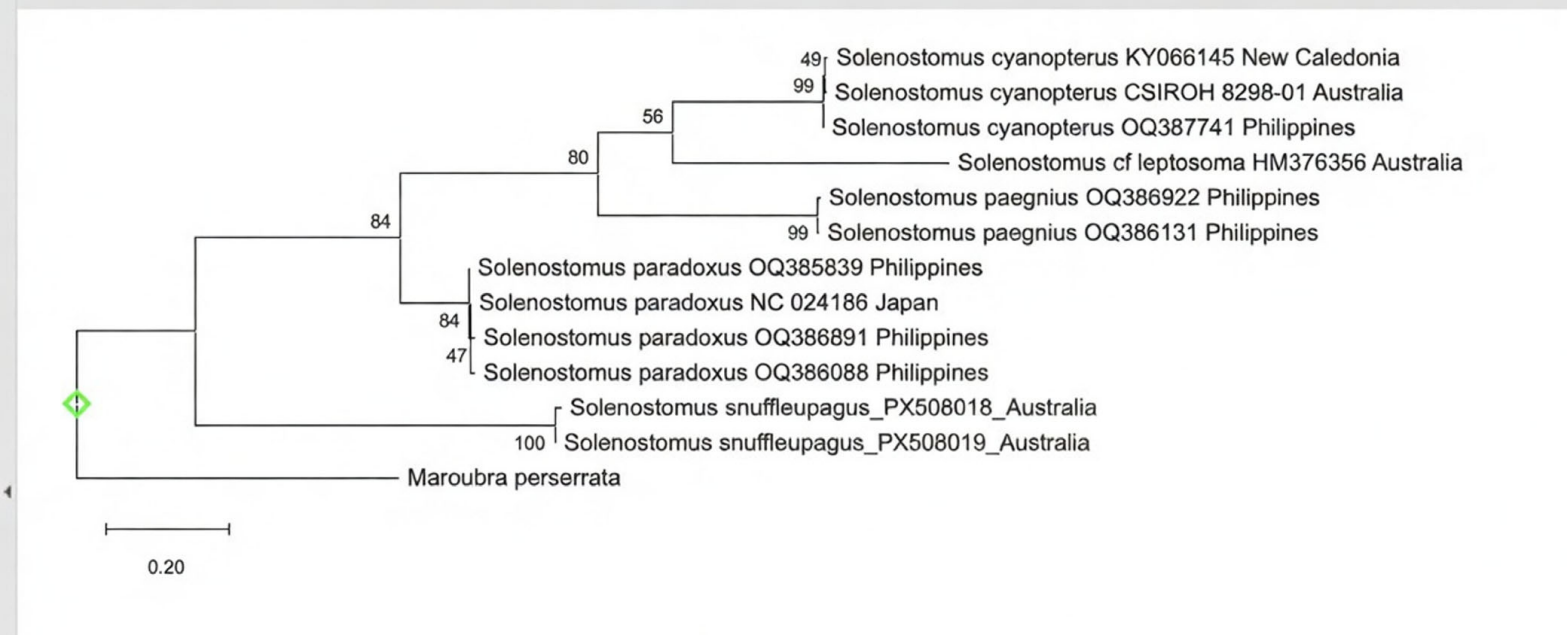
Maximum Likelihood phylogenetic tree based on partial mitochondrial COI sequences (~650 bp) of all sequenced *Solenostomus* species, rooted with the pipefish *Maroubra perserrata* (Syngnathidae) as outgroup. Inferred using the Hasegawa et al. (1985) substitution model in MEGA12; bootstrap support values (1000 replicates) are shown at nodes. Scale bar indicates nucleotide substitutions per site. *Solenostomus snuffleupagus* is recovered as the sister lineage to all remaining *Solenostomus*; the two sequenced specimens form a strongly supported clade (bootstrap 100).

DNA extraction, PCR amplification, sequence alignment and analysis of cytochrome c oxidase subunit I (COI) sequence data were conducted following the protocols of Hamilton et al. (2017). Partial COI sequences (approximately 650 bp) were obtained from two AMS non‐type specimens collected at Saxon Reef in 2022 that had been preserved in RNAlater. Available COI sequences for *Solenostomus* were retrieved from GenBank (Table 1) and used to compute uncorrected pairwise genetic distances (p‐distances) relative to the new species. All genetic distance calculations were performed using MEGA12 (Stecher et al., 2025). Newly generated sequences have been deposited in GenBank under accession numbers PX508018 and PX508019. Sequences were aligned using MUSCLE as implemented in MEGA12 (Stecher et al., 2025). The best‐fit nucleotide substitution model was selected using the Model Selection tool in MEGA12 under the Bayesian Information Criterion (BIC), which identified the Hasegawa et al. (1985) (HKY) model as optimal. Maximum Likelihood (ML) phylogenetic analysis was performed in MEGA12 using this model. The initial tree for the heuristic search was selected by choosing the topology with the superior log‐likelihood between a Neighbour‐Joining (NJ) tree and a Maximum Parsimony (MP) tree. Branch support was estimated from 1000 bootstrap replicates. Rate variation among sites was modelled using a discrete Gamma distribution (+G, parameter = 1.1001) with 58.73% of sites treated as evolutionarily invariant (+I). The alignment encompassed 13 nucleotide sequences with 676 positions; *Maroubra perserrata* was used as outgroup (Figure 1). Analyses were conducted using up to 4 parallel computing threads. Distance‐based species delimitation was performed using ASAP (Assemble Species by Automatic Partitioning; Puillandre et al., 2021) as implemented on the SPART Explorer platform (https://spartexplorer.mnhn.fr/). The aligned COI dataset was submitted with uncorrected p‐distances and default parameters. The 10 best‐scoring partitions were evaluated to assess congruence across partitioning schemes.

**TABLE 1:** Specimens of *Solenostomus* species used in molecular analyses, with voucher numbers, GenBank accession numbers, collecting localities and sequence lengths (base pairs, bp) for the mitochondrial COI gene.

Species	Voucher	GenBank	Locality	Bp
***Solenostomus snuffleupagus***	**AMS I.33751–047**	**PX508019**	**Portlock Reef, Coral Sea, Australia**	**638**
***Solenostomus snuffleupagus***	**NTM S.13600–047**	**PX508018**	**Ashmore Reef, Coral Sea, Australia**	**633**
*Solenostomus paegnius*	VERDE‐255	OQ386922	Verde Island, Philippines	655
*Solenostomus paegnius*	VERDE‐425	OQ386131	Verde Island, Philippines	655
*Solenostomus paradoxus*	VERDE‐413	OQ386891	Verde Island, Philippines	655
*Solenostomus paradoxus*	PIL‐176	OQ386088	Verde Island, Philippines	655
*Solenostomus paradoxus*	VERDE‐585	OQ385839	Verde Island, Philippines	655
*Solenostomus paradoxus*	–	NC_024186	Japan	656
*Solenostomus cyanopterus*	PIL‐294	OQ387741	Pilar, Philippines	655
*Solenostomus cyanopterus*	HH‐0759	KY066145	New Caledonia	655
*Solenostomus cyanopterus*	CSIROH 8298–01	–	Australia	676
*Solenostomus* cf. *leptosoma*	BW‐A5254	HM376356	Australia	658

*Note*: Sequences newly generated for this study are shown in bold.

## 3 RESULTS

### 3.1 Taxonomy

**Family Solenostomidae**.

**Genus Solenostomus Lacépède 1803**.

### 3.2 *Solenostomus snuffleupagus*, Short and Harasti, new species

urn:lsid:zoobank.org:pub:A6AEC42B‐B690‐41C3‐9AE7‐F86EF1869434.

**Common Name:** Hairy Ghost Pipefish, Figures 2, 3, 4, 5, 6, 7, 8, 9, 10.

**FIGURE 2:**
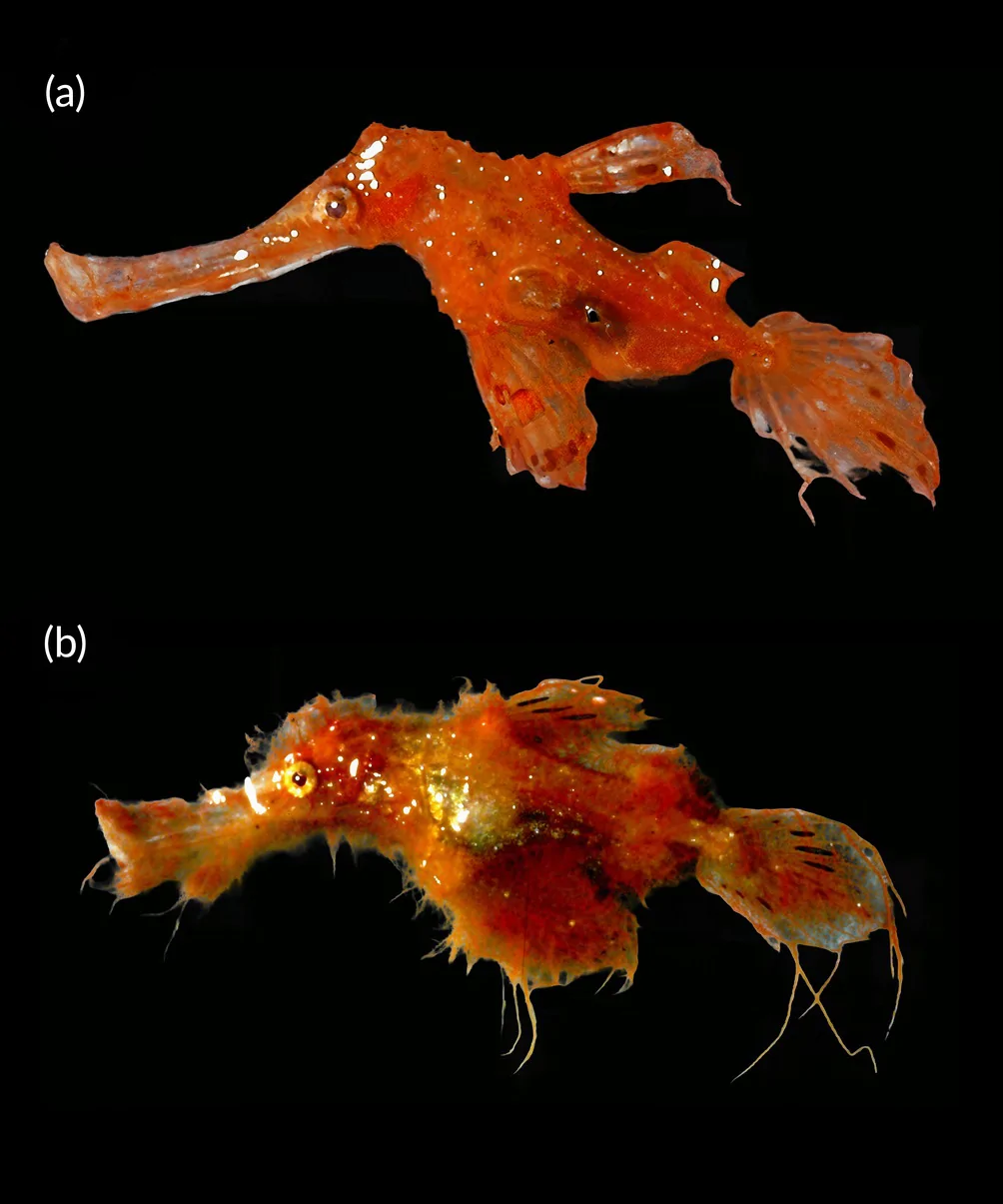
*Solenostomus snuffleupagus*, freshly collected: (a) holotype, female, AMS I.33751–047, 33.6 mm SL; (b) paratype, male, NTM S.13600‐047, 21.3 mm SL.

**FIGURE 3:**
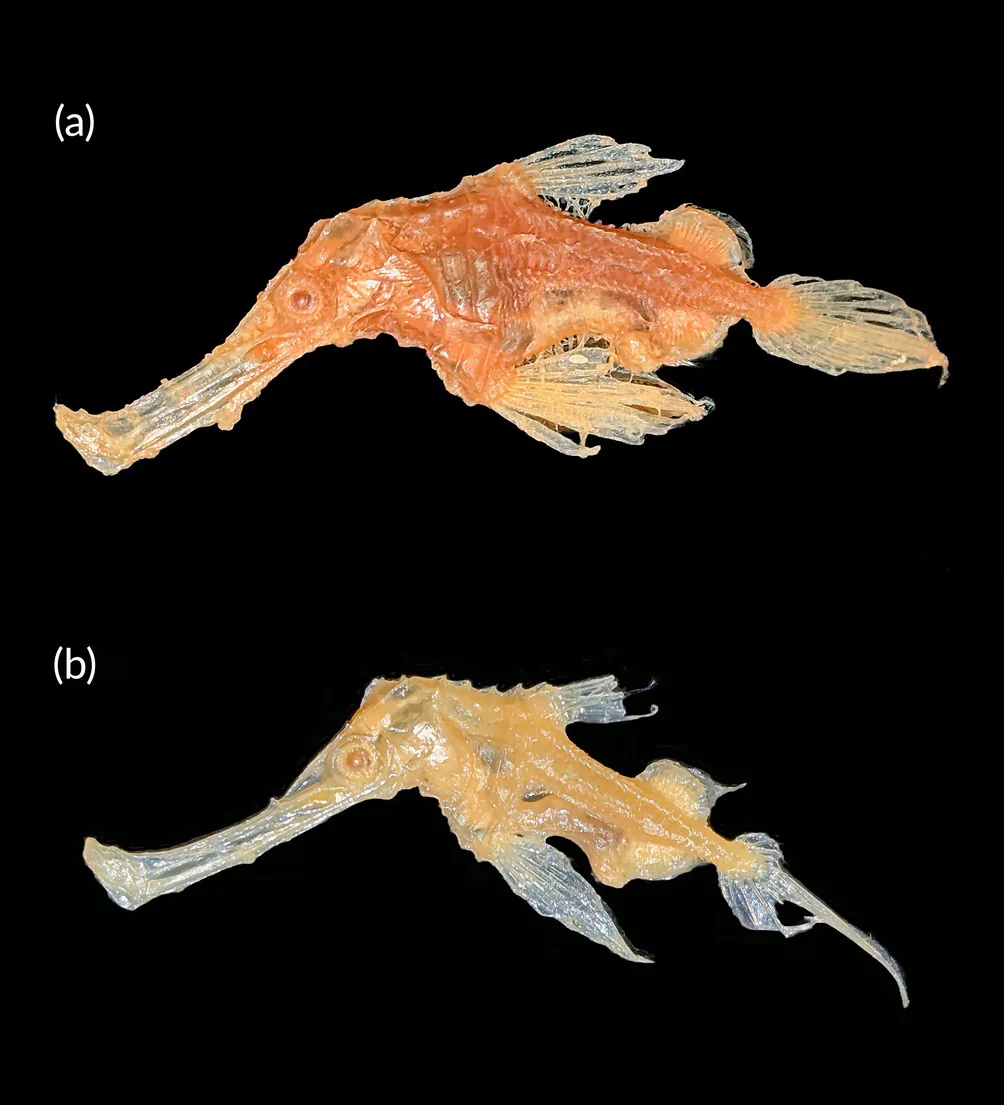
*Solenostomus snuffleupagus*, preserved specimens: (a) holotype, female, AMS I.33751–047, 33.6 mm SL; (b) paratype, male, NTM S.13600‐047, 21.3 mm SL.

**FIGURE 4:**
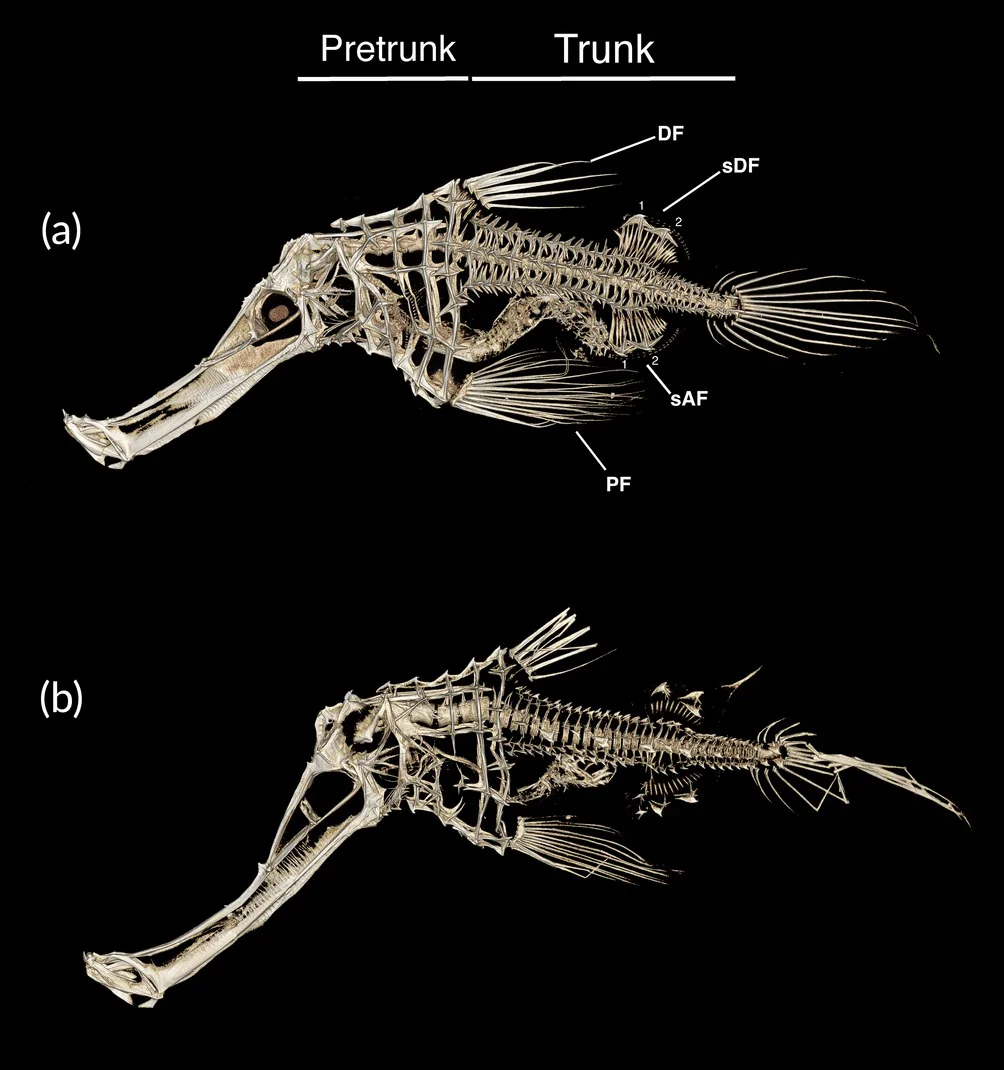
Micro‐CT reconstructions (lateral view) of *S. snuffleupagus*: (a) holotype, female, AMS I.33751‐047; (b) paratype, male, NTM S.13600–047. DF, dorsal‐fin; PF, pectoral‐fin; sAF, soft anal‐fin; sDF, soft dorsal‐fin. Numbers 1 and 2 indicate the respective pterygiophores supporting the soft dorsal‐ and anal‐fins.

**FIGURE 5:**
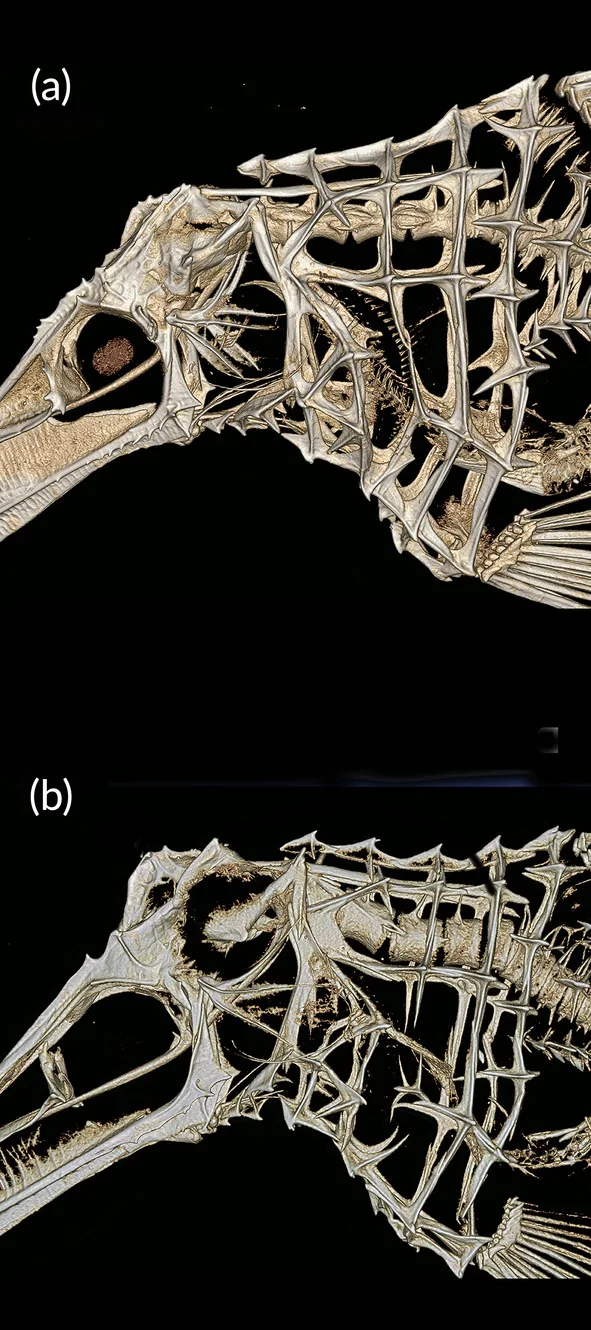
Micro‐CT reconstructions of the pretrunk and supraoccipital crest of *S. snuffleupagus*: (a) holotype, female, AMS I.33751‐047; (b) paratype, male, NTM S.13600‐047.

**FIGURE 6:**
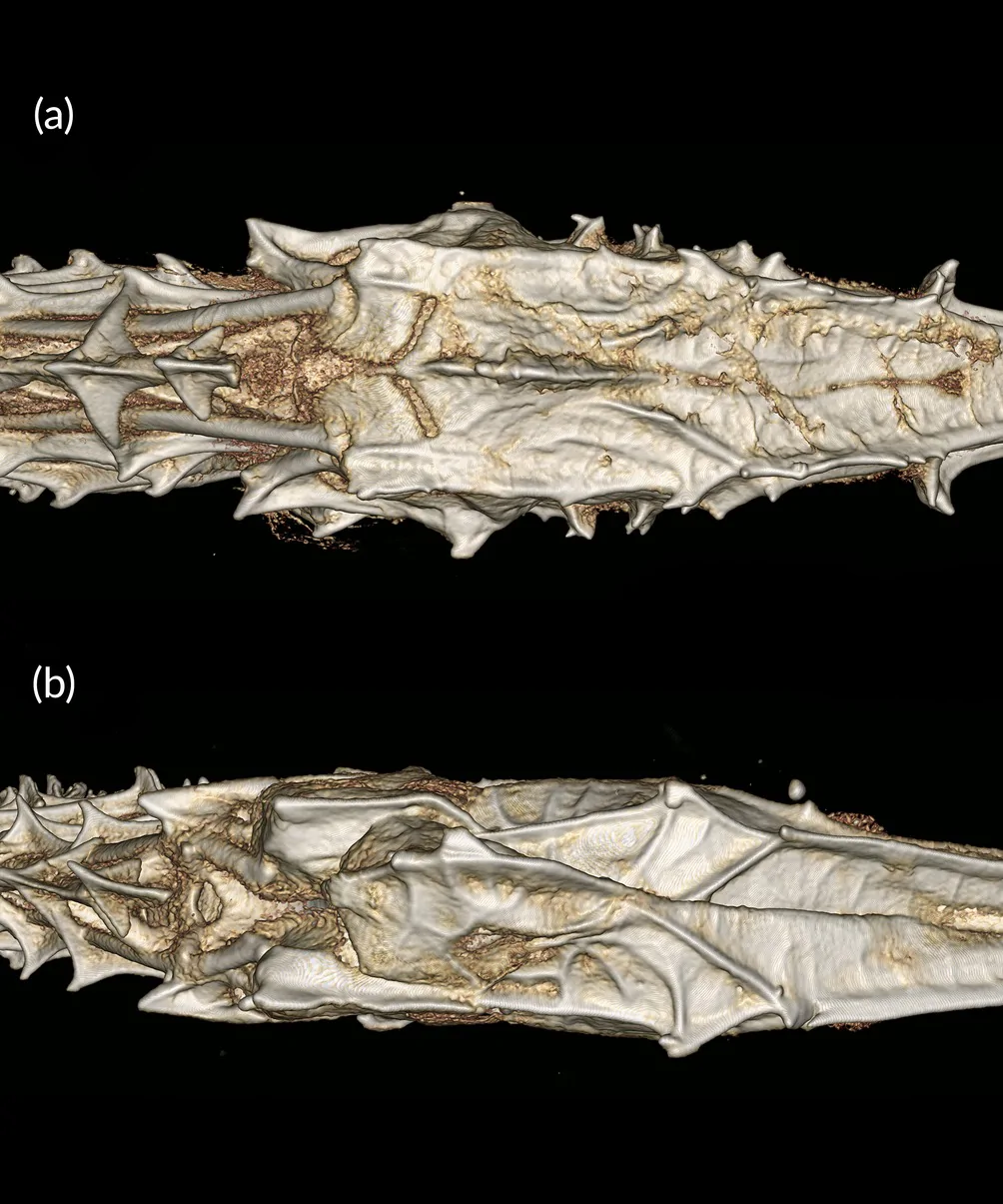
Micro‐CT reconstructions (dorsal view) of the supraoccipital crest of *S. snuffleupagus*: (a) holotype, female, AMS I.33751‐047; (b) paratype, male, NTM S.13600‐047.

**FIGURE 7:**
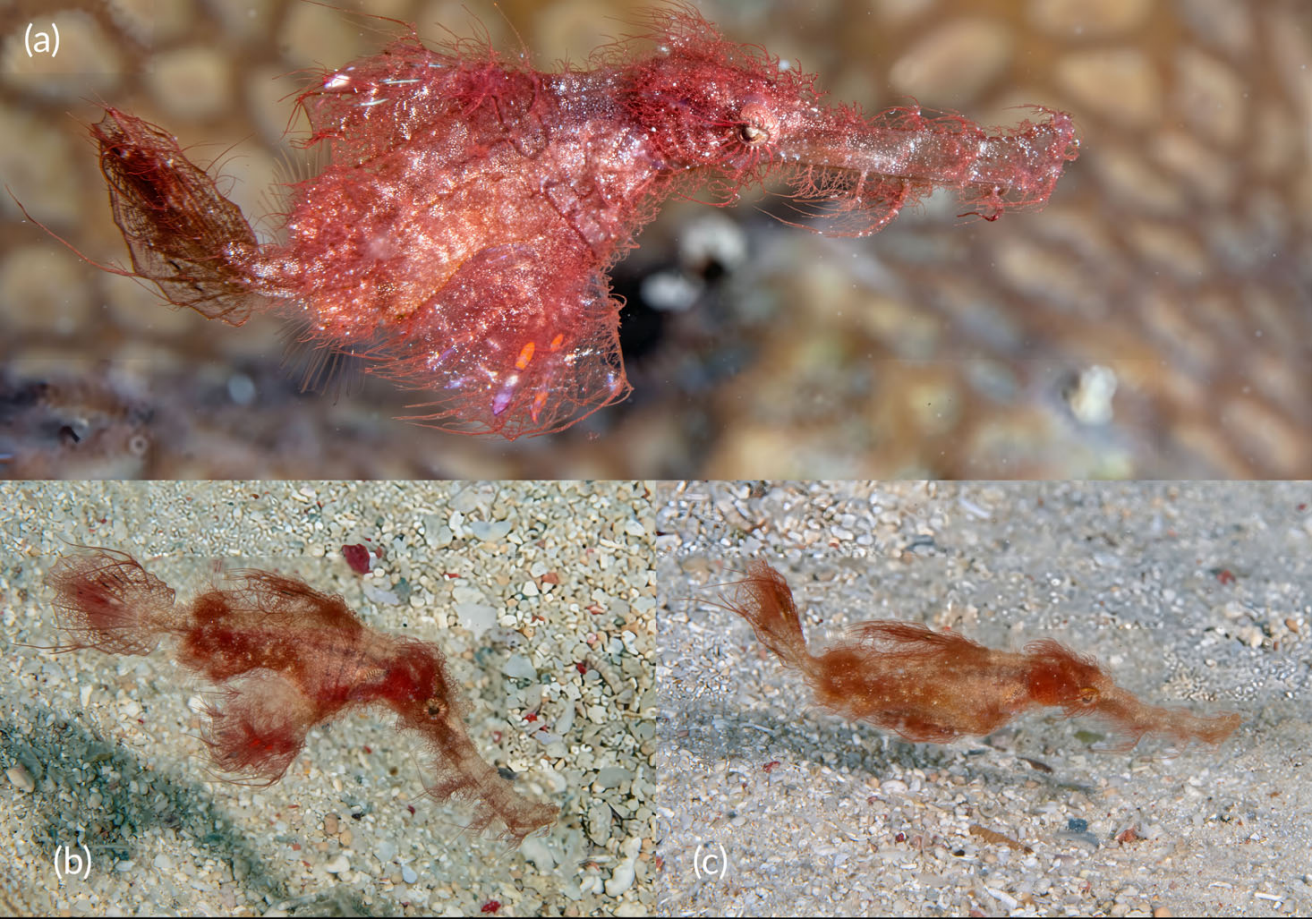
*Solenostomus snuffleupagus*, in situ, Great Barrier Reef, Australia, 2022: (a) adult individual; (b) female specimen used for genetic analysis; (c) male specimen used for genetic analysis (photographs by David Harasti).

**FIGURE 8:**
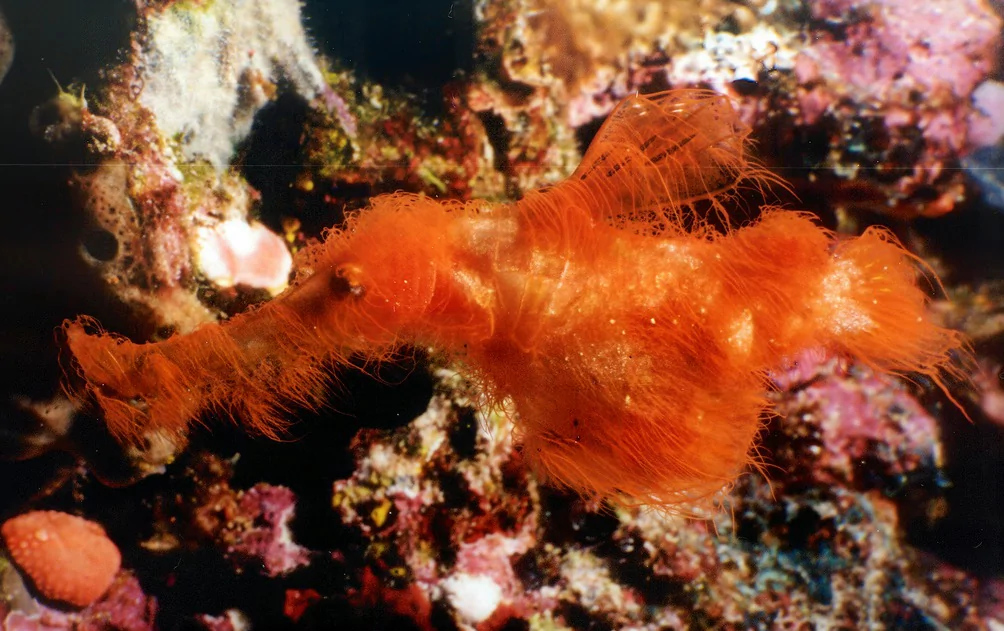
*Solenostomus snuffleupagus*, in situ, Papua New Guinea, 2003 (photograph by David Harasti).

**FIGURE 9:**
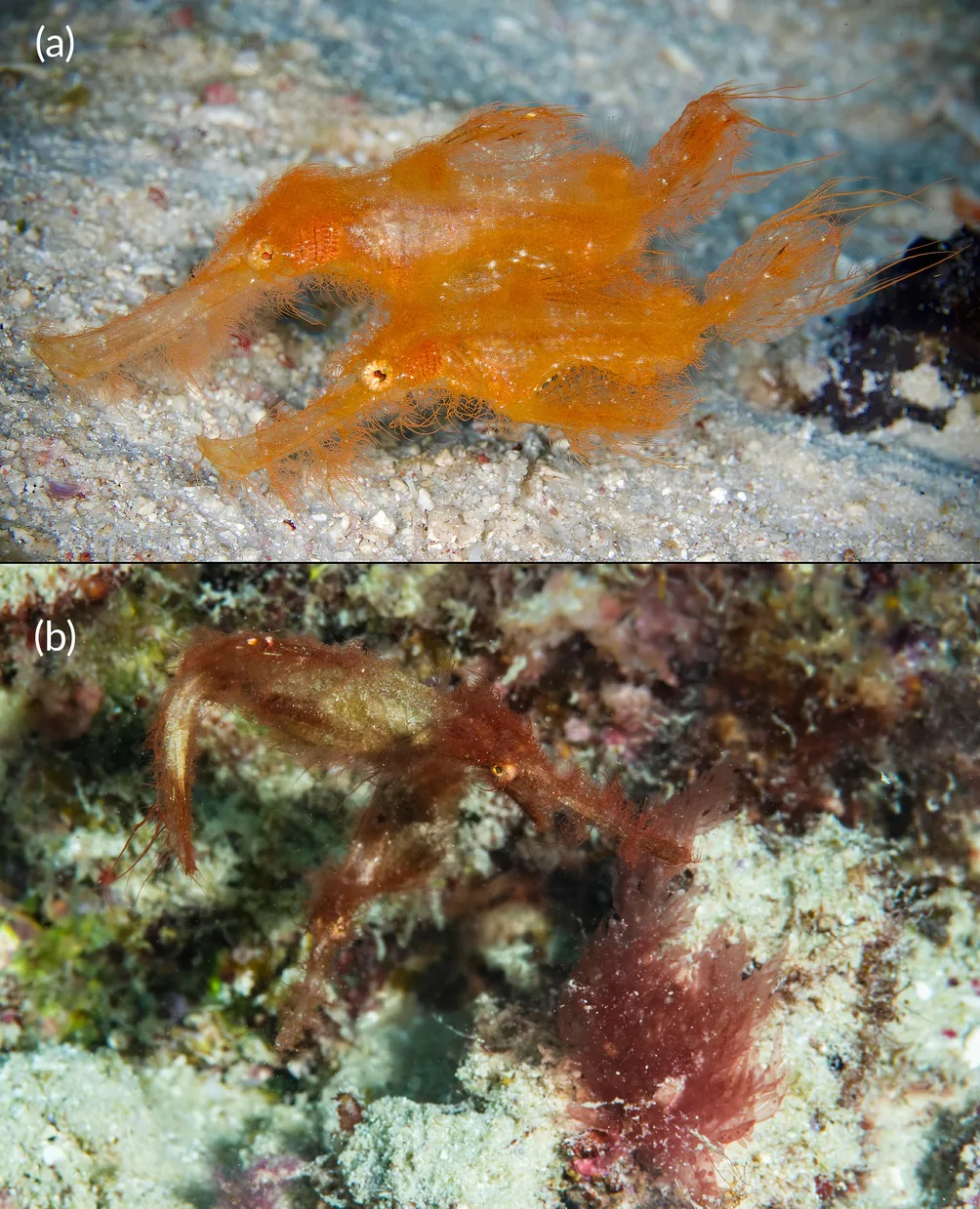
*Solenostomus snuffleupagus*, in situ, male–female pair: (a) Somosomo Strait, Fiji (photograph by Chad Cipiti @chaddashwick); (b) Great Barrier Reef, Australia (photograph by Aaron Smith). Females are positioned above the males in the water column.

**FIGURE 10:**
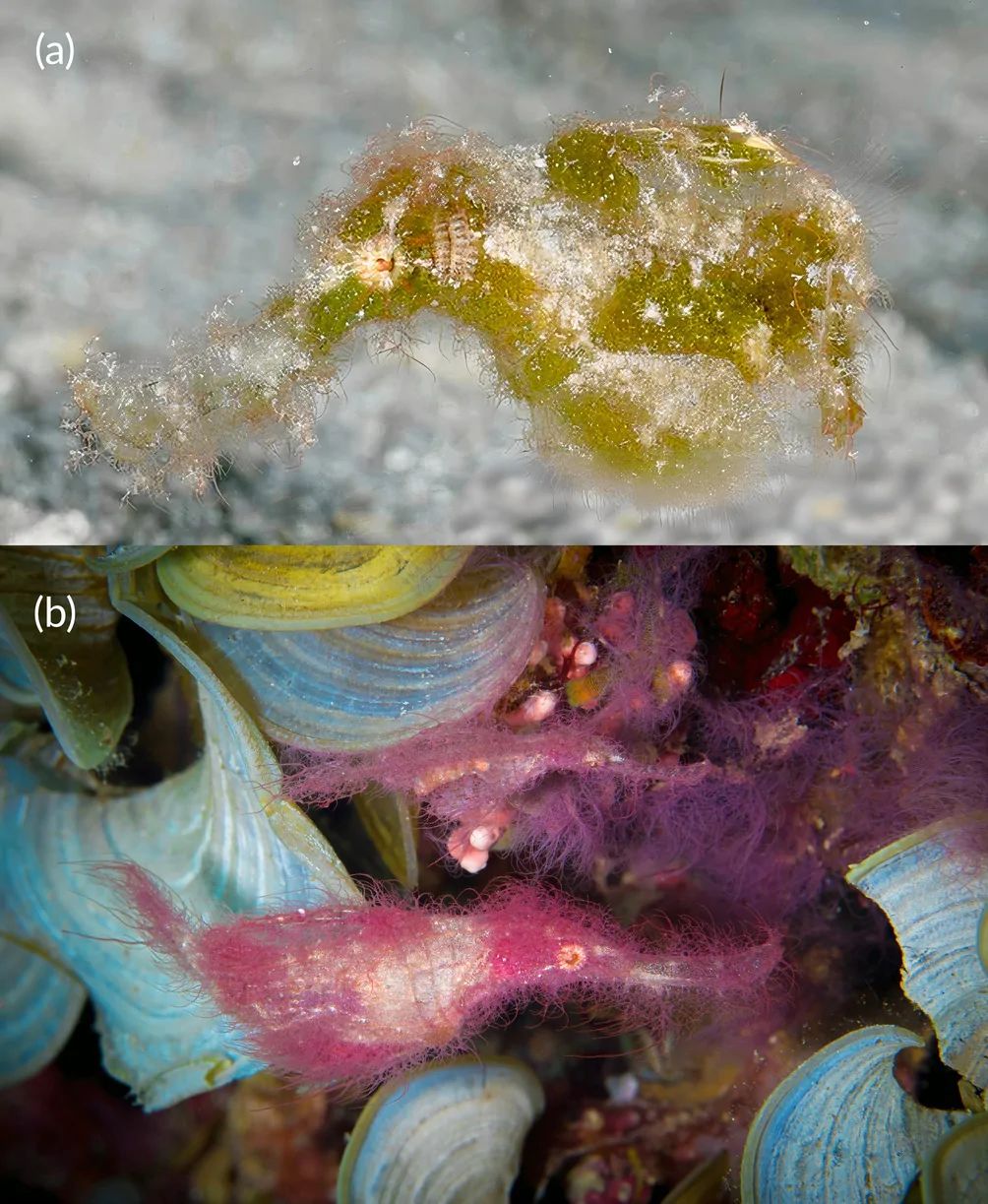
*Solenostomus snuffleupagus*, in situ, showing colour variation: (a) green individual from the Great Barrier Reef, Australia (photograph by Al Smith); (b) purple male–female pair from Papua New Guinea, with the male positioned above the female in the water column (photograph by Michael Workman @photoplatypus).

#### 3.2.1 Holotype

AMS I.33751‐047, 33.6 mm SL, female, Portlock Reef, E. side of Eastern Reef in S. group, Coral Sea, Queensland, Australia, 9°35′11″ S, 144°48′37″ E 5‐31 m depth, 29 January 1993, FNQ 93‐63 (HKL 93–37), collected by FNQ team.

#### 3.2.2 Paratype

NTM S.13600‐047 (ex AMS I.33720‐040), 21.3 mm SL, male, outer reef wall, SE Corner, Ashmore Reef, Coral Sea, Queensland, Australia, vertical coral wall, field station FNQ 93‐32 (HL 93‐17, close to HL 93‐15). Plate Corals, Sea Fans, Hydroids, Few Whips, 10°22′51.6″ S, 143°31′37.2″ E, 11–32 m depth, 19 January 1993, collected by FNQ team, identified as *Soelnostomus* [sic] sp. by H.K. Larson.

##### Comparative material

All identified as *S. paegnius* by the present authors. CAS 78331, 1 female, 54.3 mm SL, New South Wales, Australia, died in transport to Steinhart Aquarium, 2 April 1977; originally identified as *Solenostomus* sp. CAS 240194, 1 male, 35.1 mm SL, Puerto Galera Bay, Coco Beach, Oriental Mindoro, Philippines, 13°31′ N, 120°57′ E, 11.4 m depth, 12 April 2015, coll. K. A. Miller, S. Cohen, S. Bedard, E. Ong & J. Comendador. USNM 436291, 1 male, SL not measurable (caudal region damaged), Verde Island, San Agustin East, Batangas, Philippines, 13°33′ N, 121°04′ E, 12 m depth, 17 April 2015, coll. D. Catania & D. Dumale. USNM 436453, 1 female, 39.5 mm SL, off point between White Beach and Bayanan Beach, Puerto Galera, Oriental Mindoro, Philippines, 13°30′ N, 120°54′ E, 23.5 m depth, 30 April 2015, coll. D. Catania, D. Pitassy & D. Dumale. SAIAB 39239, 1 female, 45.6 mm SL, East London Shooting Range, pool, Eastern Cape, South Africa, approximately 33° S, 28° E, 1–2 m depth, 13 April 1992; originally identified as S. cyanopterus.

#### 3.2.3 Diagnosis

A species of *Solenostomus* distinguished from its congeners by the following combination of characters: body compact and vertically deep anteriorly; pretrunk deep (24.4%–29.2% SL); abundant, elongate integumentary filaments conferring a conspicuously shaggy appearance, particularly dense on snout, jaws, head and fin extremities; pretrunk ossicles are narrow and elongated, separated by deep angular interspaces, forming a distinctive compact lattice; There are total 36 vertebrae. The supraoccipital crest is elevated and rounded in females, but strongly pronounced in males. There are two modified anchor‐like ossicles spanning the pterygiophores in both the soft dorsal fin (19–20 rays) and anal fin (18–19 rays). The soft dorsal fin has 19–20 rays, the anal fin has 18–19 rays, the pectoral fin has 27 rays, the caudal fin has 15 rays.

#### 3.2.4 Description

Meristic and morphometric data for the female holotype and male paratype are presented in Table 2. The body is compressed laterally, compact and vertically deep anteriorly, tapering posteriorly to a short caudal peduncle (Figures 2, 3). Standard length 33.6 mm in holotype (female), 21.3 mm in paratype (male). The body is characterized by abundant elongate filamentous integumentary projections imparting conspicuously shaggy appearance. The filaments are especially dense on the snout (particularly ventrally), jaws, head and extremities including fins and tail. The central body portion is less adorned or nearly bare (Figures 2, 3 and 7, 8, 9, 10). Total vertebrae 36; pectoral‐fin rays 27; soft dorsal‐fin rays 19–20; soft anal‐fin rays 18–19; caudal‐fin rays 15 (Table 2).

**TABLE 2:** Meristic and morphometric data for *S. snuffleupagus* types and comparative *S. paegnius* specimens.

	*S*. *snuffleupagus*	*S*. *snuffleupagus*	*S. paegnius*	*S. paegnius*	*S. paegnius*	*S. paegnius*	*S. Paegnius*
Institution	**AMS**	**NTMS**	**CAS**	**CAS**	**USNM**	**USNM**	**SAIAB**
Voucher	**I.33751–047**	**13,600–047**	**78,331**	**240,194**	**436,291**	**436,453**	**39,239**
Type	**Holotype**	**Paratype**	**Non‐type**	**Non‐type**	**Non‐type**	**Non‐type**	**Non‐type**
Sex	Female	Male	Female	Male	Male^a^	Female	Female
SL (mm)	33.6	21.3	54.3	35.1	‐	39.5	45.6
Vertebrae	36	36	33	31	‐	31	33
Pectoral‐fin rays	27	27	28	27	27	27	26
Dorsal‐fin rays	20	19	19	18	19	18	22
Anal‐fin rays	19	18	18	17	17	16	22
Caudal‐fin rays	15	15	16	16	16	16	15
Modified anchor‐like ossicles (sDF/sAF)	2/2	2/2	3/3	3/3	‐	3/3	3/3
In % of SL							
Head length	49.7	52.6	44.7	50.8	‐	45.3	45.5
Pretrunk length	14.0	11.0	11.8	12.8	‐	10.3	14.4
Trunk length	36.6	38.3	43.5	36.5	‐	45.1	42.3
Pretrunk depth	29.2	24.4	15.4	15.4	‐	14.6	17.8
Snout length	31.6	36.2	30.4	33.9	‐	28.6	31.1
In % of pretrunk length							
Pretrunk depth	2.1	2.2	1.3	1.2	0.96	1.4	1.2
In % of trunk length							
Pretrunk length	38.2	28.8	27.1	34.9	‐	22.8	34.1
In % of head length							
Snout length	63.5	68.8	67.9	66.8	68.7	63.1	68.4
In % of snout length							
Snout depth	23.6	13.6	14.8	15.7	15.5	14.3	14.4

*Note*: Soft fin rays are from preserved specimens; modified anchor‐like ossicles are from micro‐CT reconstructions and span the pterygiophores supporting the soft dorsal (sDF) and anal (sAF) fins.

^a^ Not assessed due to the tail bitten off in specimen.

Body proportions: Pretrunk length is 4.7 mm (14.0% SL) in the holotype and 2.35 mm (11.0% SL) in the paratype. Pretrunk depth is 9.8 mm (29.2% SL) in the holotype and 5.2 mm (24.4% SL) in the paratype, forming a short, robust thoracic profile. Trunk length is 12.3 mm (36.6% SL) in the holotype, 8.16 mm (38.3% SL) in the paratype. CPL 4.8 mm (14.3% SL) in holotype, 2.51 mm (11.8% SL) in the paratype. Head length is 16.7 mm (49.8% SL) in the holotype, 11.2 mm (52.6% SL) in the paratype. Snout elongate, tubular, laterally compressed; SL is 10.6 mm (31.6% SL) in the holotype, 7.7 mm (36.2% SL) in the paratype. Snout depth is 2.5 mm (24.0% of SL) in the holotype, 1.05 mm (13.6% of SL) in the paratype; females with proportionally deeper snouts. Mouth small, terminal, slightly oblique. Premaxilla edentulous, bearing two small anteriorly directed spines.

Neurocranium: The dorsal surface of the skull has three prominent longitudinal crests: a single median crest on the supraoccipital and two shorter, parallel lateral crests (Figures 5, 6). The supraoccipital crest is sexually dimorphic, being moderately elevated and rounded in females and strongly elevated, forming a pronounced ridge in males (Figures 4, 5, 6). Epiotic large, acute, positioned immediately ventral to the posterior terminus of the associated crest (Figure 6). Mesethmoid bearing a strong lateral spine (Figure 4), variably branched, prominent in the paratype. Quadrate notably elongated, constituting the primary structural element of the anterior snout; together with the broad symplectic, forming the lateral walls of the elongated snout tube.

Axial skeleton: Vertebral column comprising 36 vertebrae (Figure 4). Three anterior vertebrae are suturally united; the first five are larger and stouter than the remainder, with the second and third especially robust. The penultimate and ultimate vertebrae are expanded into a large vertical plate supporting caudal‐fin rays. The dermal skeleton is composed of large stellate ossicles arranged in transverse and longitudinal series, forming a semi‐rigid exoskeleton. Pretrunk ossicles are the largest, forming a compact lattice with conspicuously elongated rectangular interspaces (Figures 4 and 5), imparting rigidity to the high‐bodied thoracic region. The ventral ossicle series is absent between the pretrunk and soft fins. Posterior to the pretrunk, ossicles are arranged in three vertical transverse rows, smaller and more loosely connected.

Fins: Pectoral fins with 27 rays, sexually dimorphic: significantly larger and deeper in females; more slender in males. Pelvic fins supported by thin pterygiophores, each comprising seven rays; in females modified to form a brood pouch (marsupium) through fusion with the abdomen, in males remaining free and unmodified. The first dorsal fin is composed of five unsegmented bony spines, underlain by five thick, well‐ossified pterygiophores (Figure 4). Soft dorsal fin is supported by 16 pterygiophores and 20 rays in the holotype, and 14 pterygiophores and 19 rays in the paratype. The anal fin has 15 pterygiophores and 19 rays in the holotype, and 14 pterygiophores and 18 rays in the paratype. Both the soft dorsal and anal fins possess two derived anchor‐shaped ossicles at the anterior margins, spanning the pterygiophores (Figure 4a). The caudal fin is lanceolate with 15 rays in both specimens (Figure 4).

Sexual dimorphism: *Solenostomus snuffleupagus* exhibits pronounced sexual dimorphism. Females (33.6 mm SL) are notably larger and deeper‐bodied than males (21.3 mm SL), with proportionally deeper snouts, broader body depth and larger fin bases. Pelvic fins in females are modified to form a marsupium; pectoral fins are significantly larger in females. The supraoccipital crest is more elevated and pronounced in males than in females (Figures 4, 5, 6).

#### 3.2.5 Coloration

**In life:** Based on underwater photographs from multiple localities across the species' range, *S. snuffleupagus* exhibits predominantly orange to red body coloration (Figures 7, 8, 9, 10), closely matching the hue of the filamentous red macroalgae it mimics. Adult specimens typically display bright orange or red coloration, while smaller individuals (presumed juveniles) exhibit deeper red to purplish tones (Figure 7b,c). The first dorsal fin, pectoral fins and caudal fin consistently bear three oblong patches of variable colour (orange, white or purple), representing a potentially diagnostic pattern within the genus.

Intraspecific colour variation occurs across the species' range, suggesting habitat‐specific crypsis. A single green individual—the only record of this coloration—has been documented from the GBR near Cairns, Australia (Figure 10a; photograph by Al Smith). Most documented individuals from Australia and Fiji display orange to red coloration (Figures 7, 8, 9). However, some individuals observed in Papua New Guinea exhibit purple coloration (Figure 10b). This colour polymorphism likely represents phenotypic plasticity enabling individuals to match dominant benthic algal communities in their respective microhabitats.

In preservative (70% ethanol), the head and body are pale yellow‐tan and lack distinct pigmentation. Filamentous integumentary projections persist but are flattened and reduced compared to in life. The fins are translucent to pale yellow. In the holotype, the three oblong patches interpreted as potentially diagnostic remain visible in the first dorsal and caudal fins (Figure 3).

#### 3.2.6 Distribution

**FIGURE 11:**
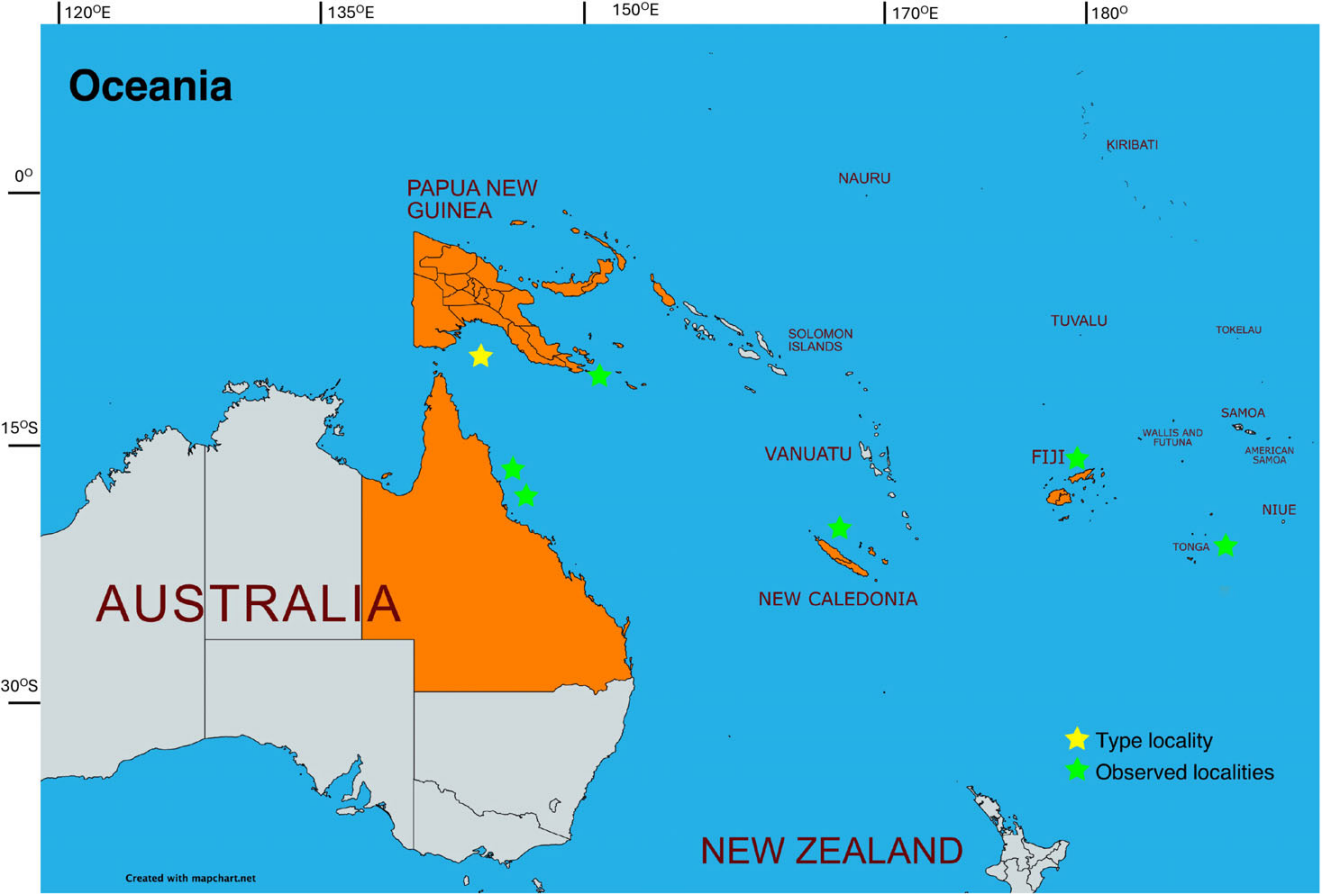
Distribution of *Solenostomus snuffleupagus* in the southwest Pacific. Yellow star indicates type locality (Portlock Reef, QLD, Australia); green stars indicate other observation localities.

*Solenostomus snuffleupagus* is currently known from coral reef habitats across the southwest Pacific (Figure 11). The type locality is Portlock Reef, on the eastern side of Eastern Reef in the southern group of the Coral Sea, Queensland, Australia (9°35′11″ S, 144°48′37″ E), positioned between the northern extent of the GBR and the reefs of Torres Strait. Additional Australian occurrences are from reef sites farther south within the GBR, including Saxon Reef, Norman Reef and Hastings Reef off Cairns, Queensland, documented through the Australasian Fishes Project on iNaturalist and through photographic submissions to the ‘GBR Macro Critters’ group (2005–present). A further observation from John Brewer Reef, located 110 km northeast of Townsville, Queensland (iNaturalist record 320,323,582), represents the southernmost verified record for the species.

Beyond Australia, confirmed occurrences have been documented from Papua New Guinea, Fiji, New Caledonia and Tonga (Figure 11; iNaturalist). Additional records from Papua New Guinea are supported by underwater photography submitted to the Facebook group ‘Ghost Pipefish’. A photograph from Papua New Guinea in 2003 (Figure 8; D. Harasti) may represent the earliest in situ photographic documentation of the species, predating all other verifiable records. A figure published by Kuiter (2000: 300–301, Figure C) under the name *Solenostomus halimeda* may represent an even earlier record; that specimen is here referred to as *S. snuffleupagus*, *a* misidentification, as the specimen is morphologically distinct from that species. Multiple observations on iNaturalist, largely identified as *S. paegnius*, confirm the presence of S. *snuffleupagus* in Fijian waters (iNaturalist observations 124,357,218, 119,688,765, 72,456,079, 42,929,265, 1,342,645 and 162,950). Two photographic records from New Caledonia submitted to iNaturalist in 2024, also identified as *S. paegnius* (iNaturalist observations 198,703,329, 196,268,974), further extend the known range of *S. snuffleupagus* into the southwestern Pacific. In Tonga, video documentation dating from 2015 has been contributed by recreational divers associated with Matafonua Lodge and Sandy Beach Resorts to the Facebook page ‘matafonualodge’.

This distribution pattern suggests occurrence throughout the periphery of the Coral Triangle, extending eastward into the broader southwest Pacific. The species appears to reach its eastern distributional limit in the Tonga Islands (https://www.facebook.com/reel/788252666016033). While observations from New Caledonia and Tonga currently represent single documented localities, this may reflect either natural range boundaries or under‐surveyed regions, rather than true rarity. The consistent presence of the species at well‐surveyed dive sites in Australia and Fiji suggests it may be more widespread than current records indicate, with distribution limited primarily by suitable habitat (coral reef with abundant filamentous macroalgae) and observer familiarity with the species.

#### 3.2.7 Depth range

Type specimens were collected at depths ranging from 5 to 31 m. Photographic records indicate their occurrence between 10 and 30 m, usually on outer reef walls and coral bommies with adjacent rubble zones.

#### 3.2.8 Habitat

Consistently associated with lower sections of coral reef bommies and adjacent rubble zones where dense growths of filamentous red macroalgae occur (Figure 9b). Specimens are typically observed hovering close to the substrate among algal filaments, exhibiting characteristic undulating movements that enhance their resemblance to drifting algae (https://www.youtube.com/watch?v=rbFhUCmwRx8).

#### 3.2.9 Biology and behaviour

*Solenostomus snuffleupagus* exhibits exceptional crypsis, closely resembling drifting filamentous algae in both morphology and behaviour. The body is densely covered with elongate filaments, creating a distinctly shaggy appearance that enhances its resemblance to algal filaments (Figure 9b).

Underwater observations indicate that specimens are most frequently encountered as solitary individuals or as male–female pairs (Figure 9), suggesting potential pair‐bonding behaviour during reproductive periods, consistent with observations of other *Solenostomus* species. Site fidelity appears strong, with one individual documented to remain in the same location for at least 6 days while actively hunting in surge‐affected waters (pers. comm. Ross Thomas, https://www.fotorosco.com, January 2025).

#### 3.2.10 Diet

Micro‐CT imaging of the holotype revealed partially digested skeletal and caudal fin remains of a small teleost (approximately 8–10 mm in length) in the gut, representing the first documented instance of piscivory in *Solenostomus* (Figure 4). The digested prey conforms to the shape of the gut, suggesting that the ingested fish was consumed whole. The genus is otherwise reported to feed primarily on small crustaceans such as mysid shrimps, caridean shrimps and zooplankton (Froese & Pauly, 2024; Kuiter, 2009). Given the ambush posture, pivot‐feeding mechanics and relatively narrow gape characteristic of ghost pipefishes, the capture of larval or juvenile fishes is biomechanically plausible and may represent opportunistic predation on prey items encountered during normal feeding behaviour.

#### 3.2.11 Etymology

The specific epithet *snuffleupagus* refers to the shaggy character Mr. Snuffleupagus, also known as ‘Snuffy’, from the children's television series Sesame Street™, in allusion to the species' distinctly shaggy, filamentous appearance and snout reminiscent of the character's covering and trunk. The name is treated as a noun in apposition (ICZN Article 31.1.2).

#### 3.2.12 Molecular divergence

Uncorrected pairwise genetic distances (p‐distances) based on partial mitochondrial COI gene sequences (~650 bp) between *S. snuffleupagus* and congeners are presented in Table 3. *Solenostomus snuffleupagus* exhibits substantial genetic divergence from all sequenced congeners: 22.0% from *S. paegnius*, 21.9% from *S. cyanopterus*, 21.0% from *S. paradoxus* and 21.8% from *S*. cf *leptosoma*. These divergence values are consistent with species‐level differentiation within Syngnathiformes and comparable to interspecific distances observed among well‐established *Solenostomus* species (Hamilton et al., 2017). The high degree of genetic divergence from *S. paegnius* (22.0%), despite superficial morphological similarity in filamentous forms, strongly supports the recognition of *S. snuffleupagus* as a distinct species. Applying a mitochondrial DNA clock rate of approximately 1.2% sequence divergence per million years for marine teleosts (Bowen et al., 2016; Reece et al., 2010), the divergence between *S. snuffleupagus* and *S. paegnius* is estimated to have occurred approximately 18.3 million years ago (early Miocene), suggesting a long independent evolutionary history.

**TABLE 3:** Uncorrected pairwise genetic distances (*p*‐distances) among *Solenostomus* species for the mitochondrial COI gene, calculated in MEGA12.

	Species	1	2	3	4	5	6	7	8	9	10	11
1	*S. snuffleupagus* (PX508019)	–	–	–	–	–	–	–	–	–	–	–
2	*S. snuffleupagus* (PX508018)	0.010	–	–	–	–	–	–	–	–	–	–
3	*S. paradoxus* (OQ386891)	0.208	0.209	–	–	–	–	–	–	–	–	–
4	*S. paradoxus* (OQ386088)	0.208	0.209	0.012	–	–	–	–	–	–	–	–
5	*S. paradoxus* (OQ385839)	0.212	0.212	0.009	0.009	–	–	–	–	–	–	–
6	*S. paradoxus* (NC_024186)	0.210	0.210	0.006	0.006	0.003	–	–	–	–	–	–
7	*S. paegnius* (OQ386922)	0.219	0.216	0.197	0.195	0.191	0.194	–	–	–	–	–
8	*S. paegnius* (OQ386131)	0.223	0.220	0.192	0.191	0.186	0.189	0.008	–	–	–	–
9	*S. cyanopterus* (OQ387741)	0.216	0.220	0.191	0.191	0.189	0.192	0.197	0.198	–	–	–
10	*S. cyanopterus* (KY066145)	0.218	0.221	0.191	0.191	0.189	0.192	0.202	0.202	0.006	–	–
11	*S. cyanopterus* (CSIROH 8298–01)	0.218	0.220	0.191	0.191	0.189	0.192	0.202	0.203	0.005	0.008	–
12	*S*. cf. *leptosoma* (HM376356)	0.218	0.218	0.205	0.200	0.203	0.203	0.202	0.198	0.177	0.174	0.173

*Note*: Values represent proportion of nucleotide differences.

ML analysis recovered *S. snuffleupagus* as the earliest‐diverging lineage within Solenostomus, placed sister to all remaining congeners (Figure 1). The two sequenced specimens (PX508018 and PX508019) formed a strongly supported monophyletic clade (bootstrap 100), with an intraspecific COI divergence of 1.0%, consistent with conspecific variation. Within the ingroup, *S. paegnius* was recovered as a sister to a clade comprising S. cyanopterus and *S*. cf. *leptosoma* (bootstrap 84), while *S. paradoxus* formed a separate clade (bootstrap 84). The recovered relationships among known congeners are broadly consistent with prior phylogenetic treatments (Hamilton et al., 2017; Stiller et al., 2022).

Distance‐based species delimitation analyses further corroborated the status of *S. snuffleupagus* as a distinct lineage. ASAP (Assemble Species by Automatic Partitioning; Puillandre et al., 2021), implemented on the SPART Explorer platform (https://spartexplorer.mnhn.fr/), recovered five species groups as the best‐scoring partition (ASAP score = 1.50, *p* = 6.2 × 10^−3^, threshold distance = 0.006), in which *S. snuffleupagus* was delimited as a cohesive unit comprising both sequenced specimens (PX508018, PX508019). The second‐ranked partition (ASAP score = 1.50, *p* = 2.9 × 10^−2^) resolved six species groups, correctly separating all five *Solenostomus* species and the outgroup. Across all 10 ranked ASAP partitions, *S. snuffleupagus* was recovered as a distinct lineage in nine; the sole exception was a two‐group partition that recognized only ingroup versus outgroup. Complementary analysis using ABGD (Automatic Barcode Gap Discovery; Puillandre et al., 2012) identified a clear barcode gap between intraspecific (0%–1.2%) and interspecific (17.3%–22.3%) distances. ABGD consistently returned six species groups across all 10 prior maximal distance values (*p* = 0.001–0.100), in both initial and recursive partitions, corresponding to the five recognized *Solenostomus* species plus the outgroup (*Maroubra perserrata*). The congruence between distance‐based delimitation methods (ASAP, ABGD) and the ML phylogenetic analysis, combined with the substantial interspecific divergence (≥20% from all congeners) and low intraspecific variation (1.0%), provides robust molecular support for the recognition of *S. snuffleupagus* as a valid species.

### 3.3 Morphological comparisons

**FIGURE 12:**
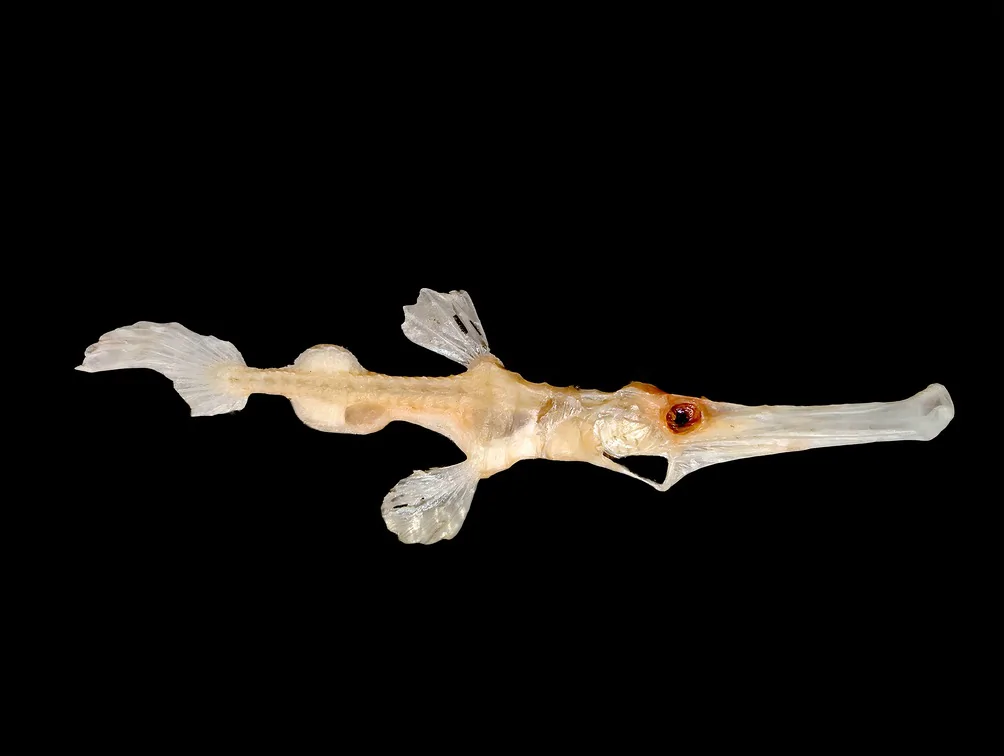
*Solenostomus paegnius*, CAS 240194, male, 35.1 mm SL, Puerto Galera Bay, Philippines.

**FIGURE 13:**
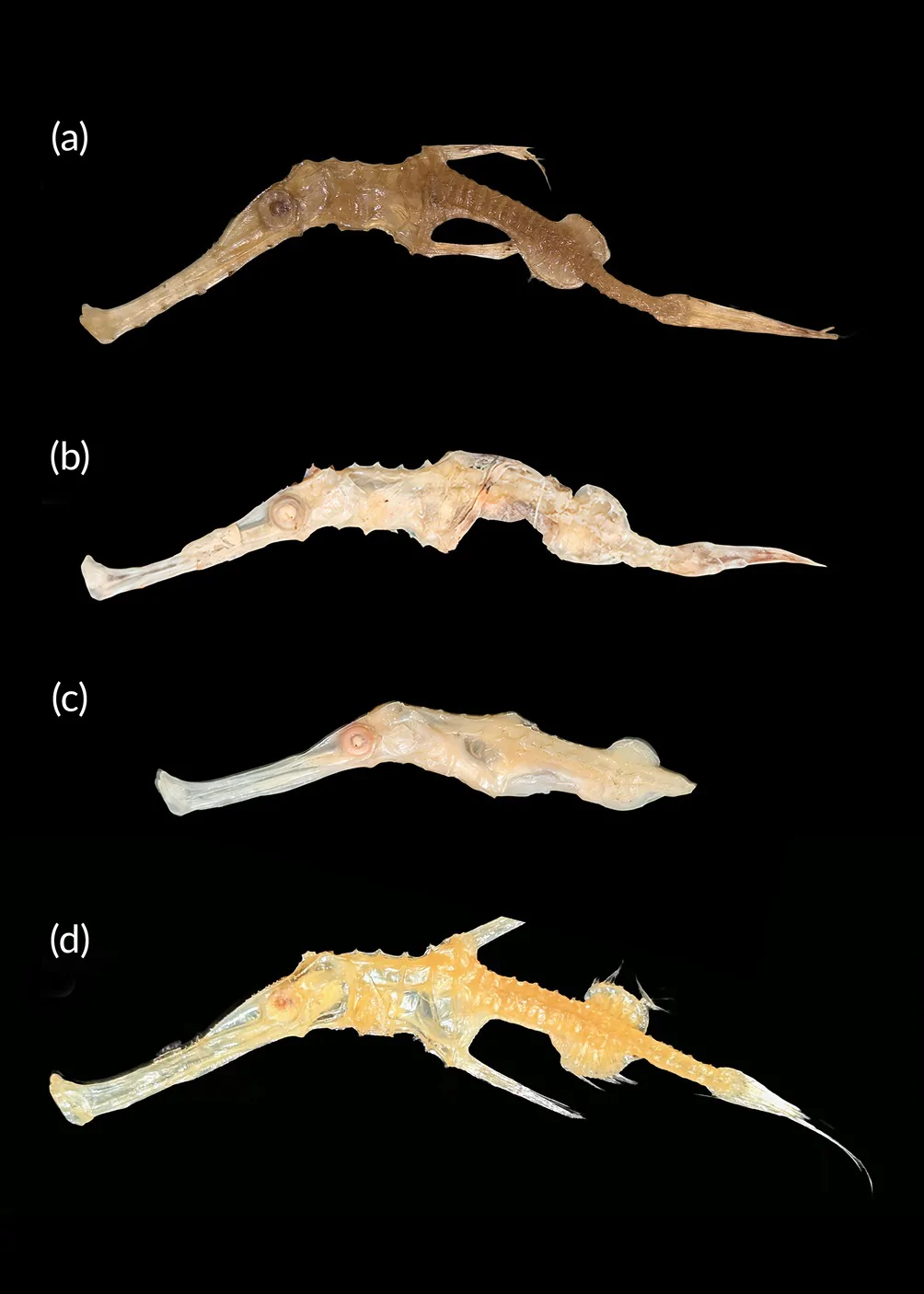
*Solenostomus paegnius*, preserved specimens, lateral view: (a) male, CAS 240194; (b) female, USNM 436453; (c) male, USNM 436291; (d) female, SAIAB 39239. CAS and USNM material from the Philippines; SAIAB specimen from South Africa.

*Solenostomus snuffleupagus* most closely resembles *S. paegnius* Jordan & Thompson, 1914, sharing a slender snout (not expanded dorsally or ventrally) and filamentous ornamentation to varying degrees. Both species have been confused in collections (e.g., the SAIAB specimen of S. paegnius was originally identified as S. cyanopterus) and in citizen science records, as some *S. paegnius* individuals exhibit elongate dermal filaments and appear ‘hairy’. However, *S. snuffleupagus* is extensively covered with elongate dermal filaments over nearly the entire body surface, imparting a conspicuously shaggy appearance (Figures 2, 3 and 7, 8, 9, 10), whereas *S. paegnius* typically exhibits only three to four discrete clusters of filaments ventral to the snout (Figures 12, 13 and 18), with few individuals showing scattered filaments elsewhere. This dense, continuous filamentation provides a reliable external characteristic distinguishing *S. snuffleupagus* from *S. paegnius* and all other congeners (absent or sparse in *S. cyanopterus*, *S. paradoxus*, *S. leptosoma*, *S. halimeda* and *S. armatus*; Orr & Fritzsche, 1993; Orr et al., 2002).

Morphometrically, *S. snuffleupagus* differs from *S. paegnius* in having a markedly deeper pretrunk (pretrunk depth 24.4%–29.2% SL vs. 14.6%–17.8% SL; no overlap). Pretrunk length overlaps broadly (11.0%–14.0% SL vs. 10.3%–14.4% SL; note the SAIAB specimen at 14.4% SL exceeds the maximum of the new species) and is therefore not diagnostic alone. With the exception of pretrunk depth, morphometric characters broadly overlap between the two species and the diagnosis rests on the combination of vertebral count (36 vs. 31–34), integumentary filaments, osteology and molecular divergence rather than on morphometrics alone. *Solenostomus snuffleupagus* further differs from all remaining congeners in possessing abundant filamentous integument (absent or sparse in *S. cyanopterus*, *S. paradoxus*, *S. leptosoma*, *S. halimeda* and *S. armatus*) and 36 total vertebrae (vs. 32–34 in all others; Orr & Fritzsche, 1993; Orret al. 2002).

**FIGURE 14:**
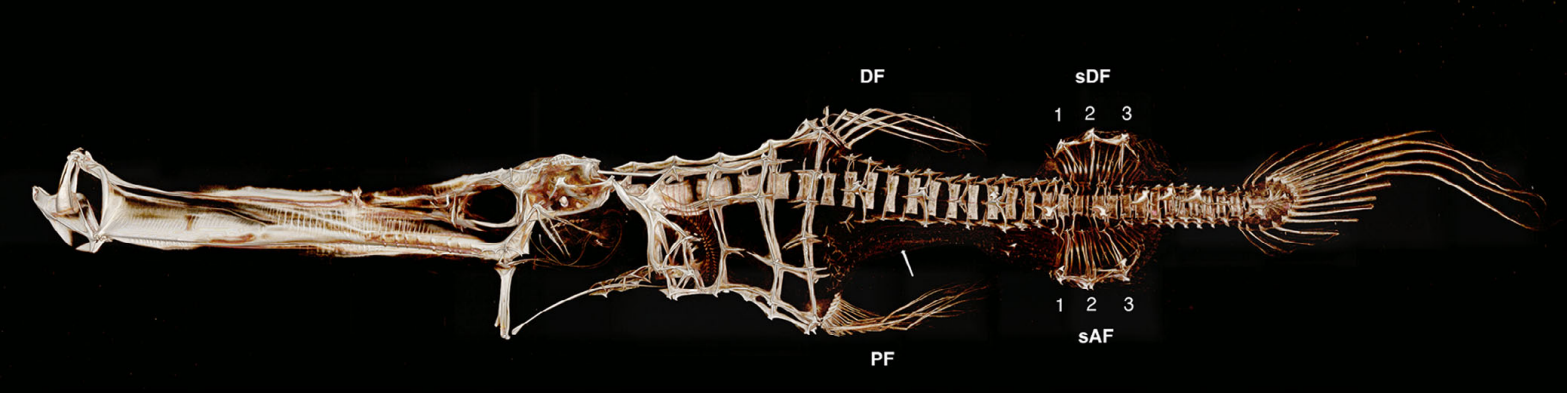
Micro‐CT reconstruction (lateral view) of CAS 240194. DF, dorsal‐fin; PF, pectoral‐fin; sAF, soft anal‐fin; sDF, soft dorsal‐fin. Numbers 1, 2 and 3 indicate the respective pterygiophores supporting the soft dorsal‐ and anal‐fins. White arrow indicates a series of small ossicles ventral to the vertebral column between the pretrunk and soft‐fins, forming a single layer of small elements in this region.

**FIGURE 15:**
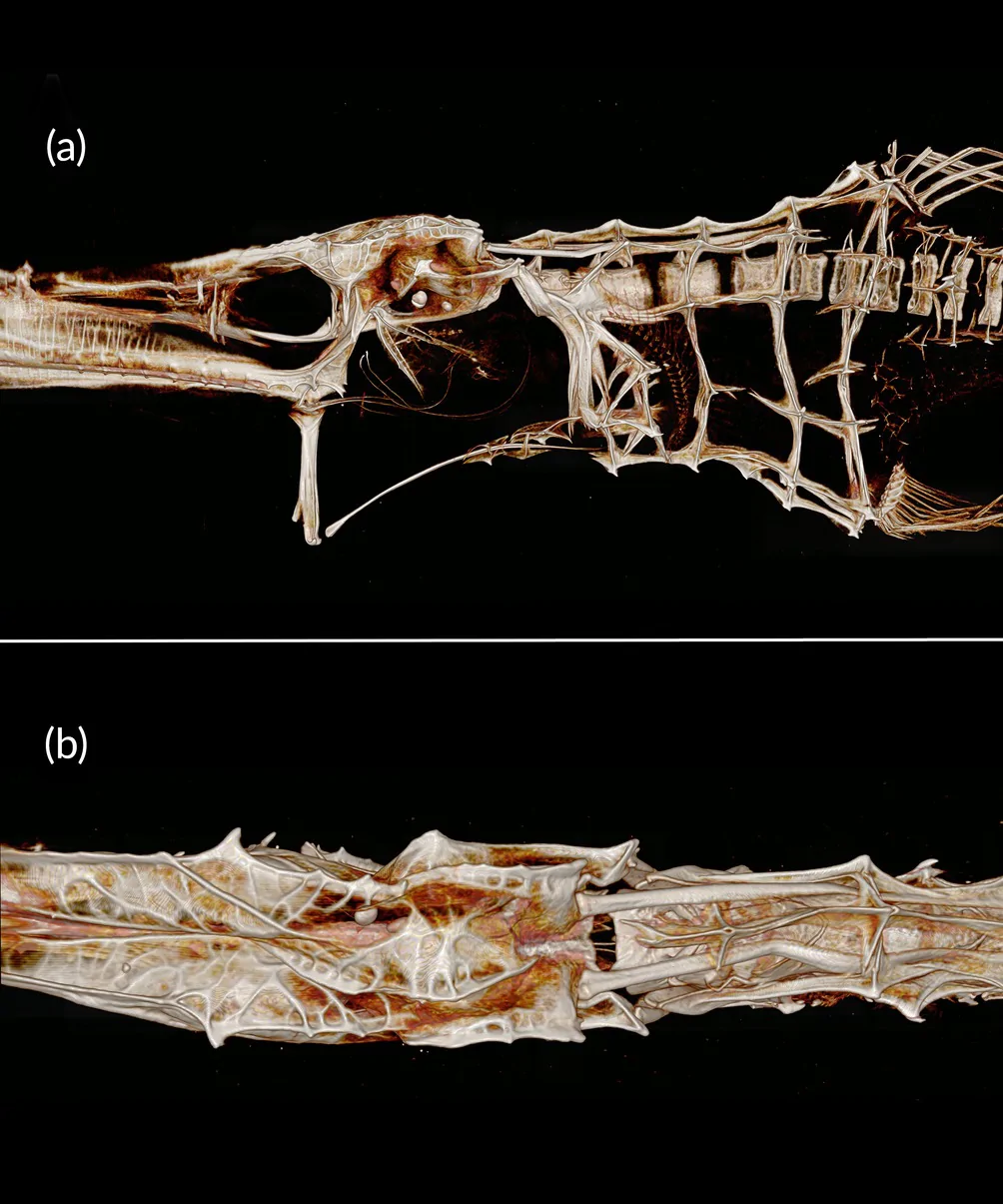
Micro‐CT reconstruction of the supraoccipital crest of CAS 240194: (a) lateral view; (b) dorsal view.

**FIGURE 16:**
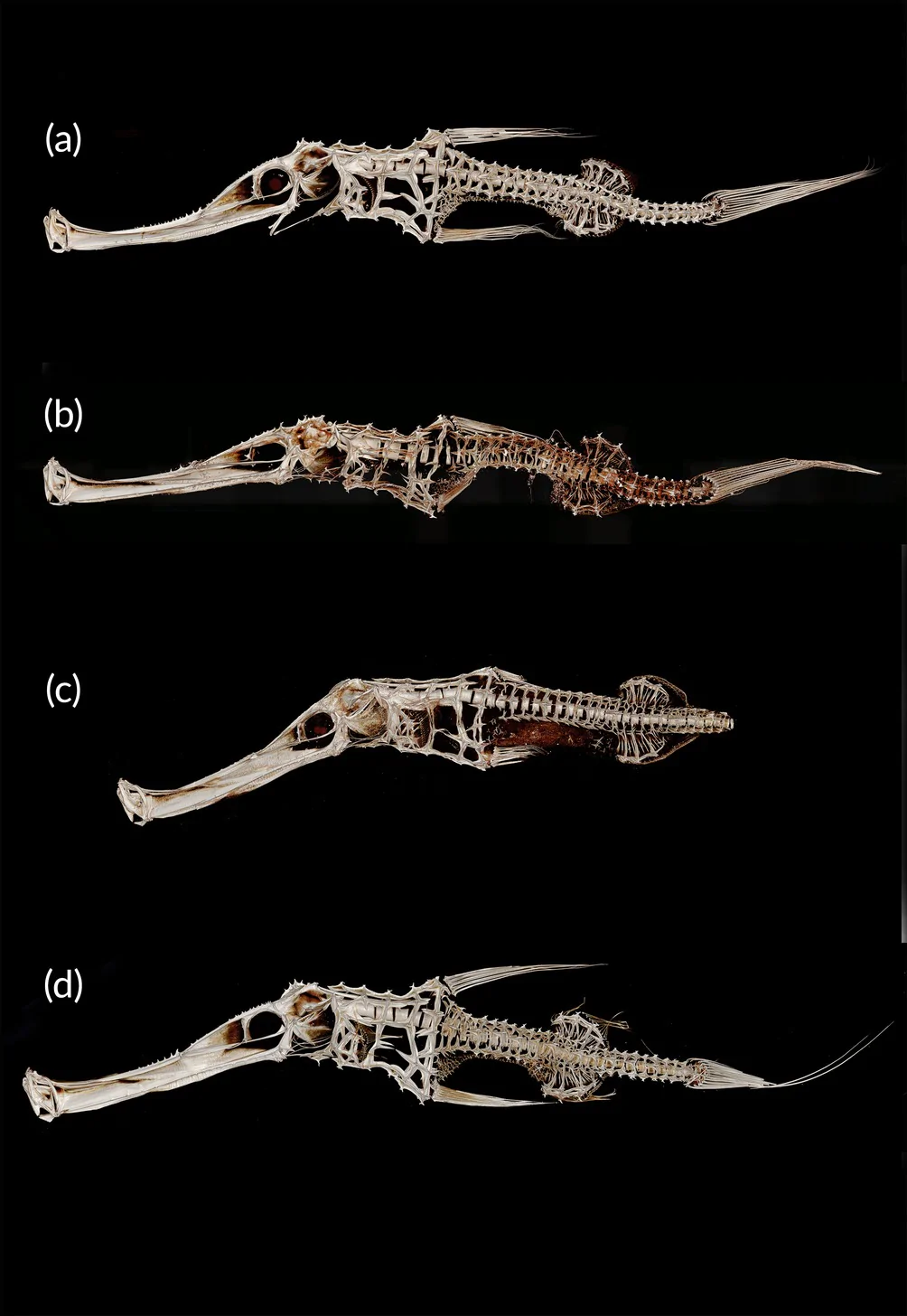
Micro‐CT reconstructions of *Solenostomus paegnius* preserved specimens, lateral view: (a) male, CAS 240194; (b) female, USNM 436453; (c) male, USNM 436291; (d) female, SAIAB 39239. CAS and USNM material from the Philippines; SAIAB specimen from South Africa.

**FIGURE 17:**
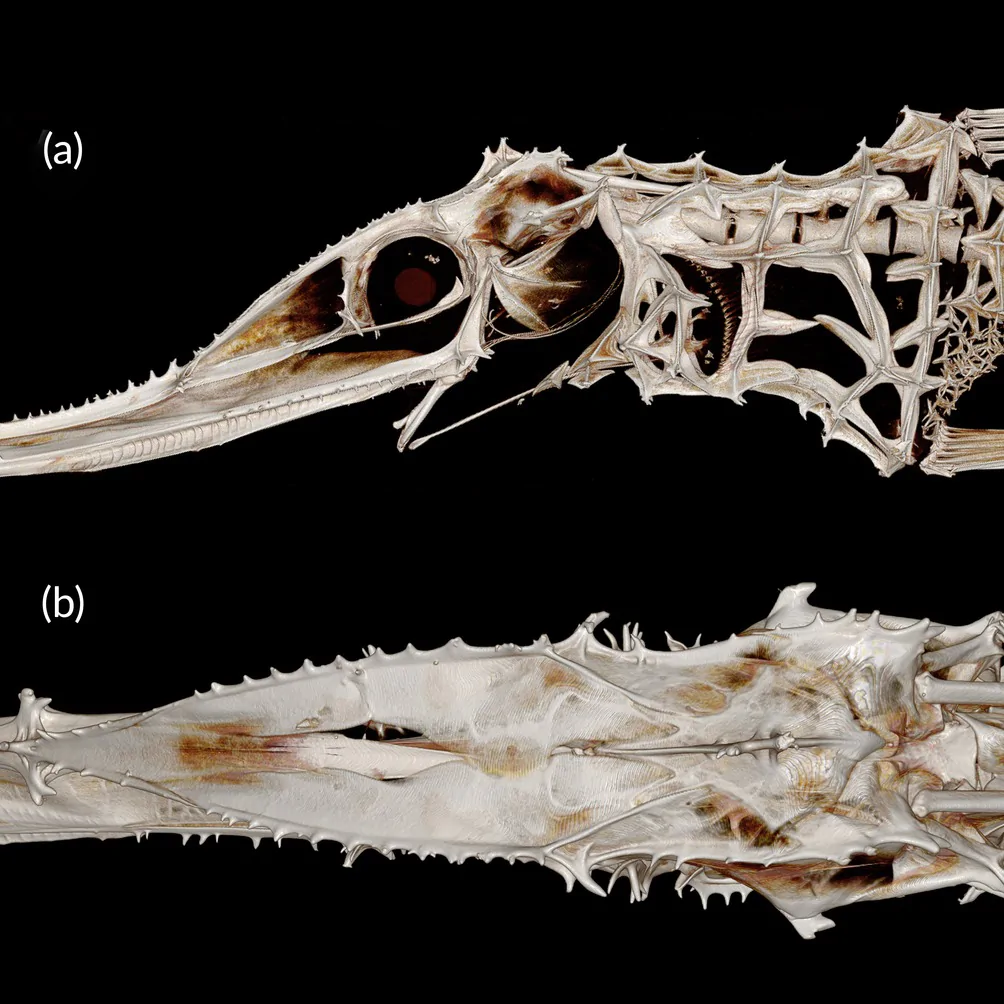
Micro‐CT reconstruction of the supraoccipital crest of *Solenostomus paegnius*, female, CAS 78331: (a) lateral view; (b) dorsal view.

Osteologically, *S. snuffleupagus* differs from *S. paegnius* in several key respects. The pretrunk region is shorter and more compact, with shallow angular interspaces between ossicles (Figures 4 and 5), contrasting with deeper rectangular interspaces in *S. paegnius* (Figures 14, 15 and 16). The supraoccipital crest is sexually dimorphic in both species but expressed oppositely: males of *S. snuffleupagus* possess a prominently elevated crest (Figure 6b), whereas females exhibit a lower, smoother crest (Figure 6a); in *S. paegnius*, the crest is strongly elevated and denticulated (bearing three to four small anterior spines) in females (Figure 17) but low and non‐denticulated in males (Figure 14). The epiotic bones are blunt and rounded dorsally in *S. snuffleupagus*, differing from the sharply pointed condition in *S. paegnius*. Vertebral counts are higher in *S. snuffleupagus* (36 total) than in *S. paegnius* (31–33; Table 2; up to 34 per Orr & Fritzsche, 1993) and all other congeners (32–34; Orr & Fritzsche, 1993; Orr et al., 2002).

#### 3.3.1 Soft dorsal and anal fin supports

Soft dorsal fin with 19–20 rays; soft anal fin with 18–19 rays (Table 2). Two modified anchor‐like ossicles span the pterygiophores in each fin (Figure 4), a configuration unique among examined Solenostomus. In *S. paegnius*, three such anchor‐like ossicles are present in each fin (Figure 14; Table 2), with soft ray counts overlapping (sDF 18–22, sAF 16–22). Soft ray counts broadly overlap with other congeners (Jordan & Thompson, 1914; Orr et al., 2002; Orr & Fritzsche, 1993) and are non‐diagnostic alone; the reduced number of anchor‐like ossicles (2 vs. 3) combined with abundant filaments and 36 vertebrae provides robust separation from *S. paegnius* and distinction from all congeners.

A further distinction involves the ventral ossicle series between the pretrunk and soft dorsal/anal fins. *Solenostomus snuffleupagus* completely lacks this series (Figure 4), whereas *S. paegnius* possesses a single row of small, simple ossicles forming a narrow ventral band beneath the vertebral column (Figure 14, white arrow). These osteological characters, together with opposing supraoccipital crest dimorphism, compact body form and extensive filamentation, serve as reliable diagnostic differences between the two species.

**FIGURE 18:**
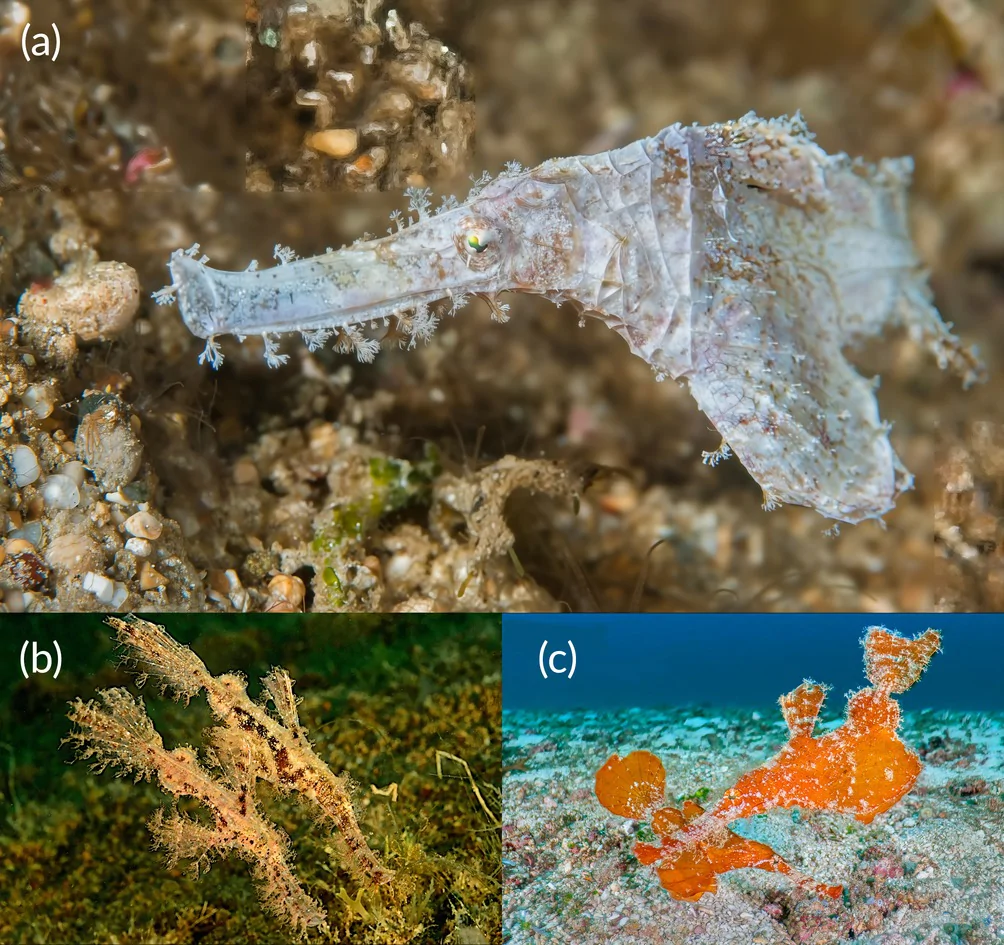
*Solenostomus paegnius*, in situ: (a) Indonesia (photograph by Richard Smith); (b) Philippines, male–female pair with the female positioned above the male in the water column (photograph by Lynn Funkhouser); (c) Philippines, male–female pair with the female positioned above the male in the water column (photograph by Alex Tyrrell @dive4photos).

Morphometrically, *S. snuffleupagus* has a pretrunk length of 11.0%–14.0% SL (overlapping with 10.3%–14.4% SL in *S. paegnius*) and a substantially deeper pretrunk (pretrunk depth 24.4%–29.2% vs. 14.6%–17.8% in *S. paegnius*; Table 2). The pretrunk length to trunk length ratio averages 0.28–0.38 in *S. snuffleupagus* (reflecting its compact, vertically deep thoracic profile) versus 0.23–0.35 in *S. paegnius* (more elongate and slender). With the exception of pretrunk depth (pretrunk depth as % SL), these proportional characters show considerable overlap between the two species. Collectively, these proportional, osteological and external differences clearly distinguish S. *snuffleupagus* from *S. paegnius* and all other congeners. In situ, *S. paegnius* typically presents a sleek profile with sparse filamentous ornamentation (Figure 18a,c), although some individuals exhibit denser filaments (Figure 18b), underscoring the potential for misidentification with *S. snuffleupagus* (Figures 7, 8, 9, 10) and the importance of integrative diagnosis beyond external appearance alone.

## 4 DISCUSSION

The systematics of *Solenostomus* have historically been shaped by foundational morphological studies, particularly Jungersen (1910), who provided detailed accounts of external and skeletal morphology. Later, Orr and Fritzsche (1993) refined species distinctions based on meristic and morphometric analyses of museum specimens, and Orr et al. (2002) further clarified the identities of *S. cyanopterus* and *S. paradoxus* with the description of *S. halimeda*. Despite these advances, *S. paegnius* remained poorly defined and unexamined in recent works, and filamentous forms have continued to challenge species‐level resolution. It should be noted that *S. paegnius* itself requires a modern comprehensive systematic revision with examination of all type material; the specimens identified as *S. paegnius* in this study represent our best determination based on available material. This study resolves this long‐standing ambiguity through the formal description of *S. snuffleupagus*, integrating osteological and molecular data to clarify species boundaries within this morphologically variable genus.

Across all examined materials, no intermediate morphologies or sympatric co‐occurrences were documented between *S. snuffleupagus* and *S. paegnius*, suggesting reproductive isolation and supporting their recognition as distinct species. The distribution of *S. snuffleupagus* in the Southwest Pacific region may reflect limited sampling effort rather than true range restriction, given that suitable filamentous red algal habitats occur widely across the Indo–West Pacific. In contrast, *S. paegnius* occupies a broader range of benthic environments throughout the Indo–West Pacific.

This study is also the first taxonomic account of *Solenostomus* to employ high‐resolution micro‐CT scanning to examine subtle osteological characters, including the configuration of the supraoccipital crest, epiotic processes, pretrunk ossicle arrangement and ventral ossicle layering. CT data were used both qualitatively and quantitatively to visualize three‐dimensional ossicle topology, fin‐ray bases and crest height. This non‐destructive approach enabled a detailed assessment of fragile type and comparative material, yielding new morphological insights while preserving specimen integrity. The integration of these data with traditional meristic and molecular analyses provides a powerful framework for resolving long‐standing taxonomic ambiguities and exemplifies the growing role of digital morphology in the systematic study of rare and delicate syngnathiform fishes. Sexual dimorphism in cranial and fin morphology occurs across the genus, yet the reversed pattern of supraoccipital crest development between the sexes in *S. snuffleupagus* and *S. paegnius*—elevated in males of the former but in females of the latter—provides an additional taxonomically informative character.

Recognition of *S. snuffleupagus* as distinct also underscores the ecological importance of coral‐associated and algal habitats as reservoirs of cryptic syngnathiform diversity, warranting further targeted surveys across the Coral Sea, western Pacific and Indian Ocean margins.

The formal description of *S. snuffleupagus* is based on two type specimens (female holotype and male paratype) plus two non‐type individuals used for molecular analysis. While a larger type series is often desirable, the diagnosis is robustly supported by an integrative approach combining distinctive external morphology, osteology and mitochondrial COI sequences (uncorrected p‐distance 22.0% from *S. paegnius*). The conspicuous shaggy appearance conferred by abundant elongate filaments is consistently observed across the species' known range in numerous high‐resolution in situ photographs (Figures 7, 8, 9, 10), providing additional confidence in species delimitation despite limited preserved material.

Searches of major ichthyological collections (Australian Museum [AMS], California Academy of Sciences [CAS], United States National Museum [USNM], Northern Territory Museum [NTM], SAIAB and Bishop Museum [BPBM]) yielded no additional specimens of *S. snuffleupagus* beyond those described here. This scarcity in museum holdings is attributed to the species' extreme crypsis, small adult size and occurrence in complex reef habitats with dense filamentous red macroalgae that are difficult to sample systematically, rather than true rarity in the wild. The same pattern holds for the morphologically similar *S. paegnius*, which has a wider reported Indo‐West Pacific distribution but similarly limited museum material due to its cryptic habits and association with filamentous algae (Orr & Fritzsche, 1993; diver observations in muck habitats [pers. obs.]). The modest type series is justified by the inherent challenges of collecting and preserving highly cryptic syngnathiforms. The availability of both sexes has enabled the description of pronounced sexual dimorphism and molecular data have provided independent evidence confirming the validity of the species. In conclusion, the formal description of *S. snuffleupagus* brings the number of recognized ghost pipefish species to seven and highlights the continued potential for discovery of cryptic diversity within this genus. The species' southwestern Pacific distribution, specialized habitat association with filamentous red macroalgae, and apparent rarity in museum collections underscore the importance of integrating citizen science observations with traditional taxonomic methods. The growing archive of underwater imagery on platforms such as iNaturalist and dedicated Facebook groups has proven instrumental in documenting the geographic range and colour variation of *S. snuffleupagus*, compensating for the inherent difficulty of collecting these highly cryptic fishes. Future molecular sampling across the species' range, combined with targeted surveys of shallow reef habitats where filamentous algae predominate, will be essential for understanding population connectivity and assessing conservation status. This study demonstrates that even well‐surveyed regions such as the GBR continue to harbour undescribed syngnathiform diversity, warranting sustained taxonomic attention to this charismatic but poorly understood group.

### MATERIAL EXAMINED

*Solenostomus snuffleupagus* Short & Harasti, new species.

**Holotype**. AMS I.33751‐047, 33.6 mm SL, female, Portlock Reef, Eastern side of Eastern Reef in Southern group, Coral Sea, Queensland, Australia, 9°35′11″ S, 144°48′37″ E, 5–31 m, 29 January 1993, FNQ team. Used for morphometrics, micro‐CT.

**Paratype**. NTM S.13600‐047 (ex AMS I.33720–040), 21.3 mm SL, male, outer reef wall, SE Corner, Ashmore Reef, Coral Sea, Queensland, Australia, 10°22′51.6″ S, 143°31′37.2″ E, 11–32 m, 19 January 1993, FNQ team. Used for morphometrics, micro‐CT.

**Non‐type material**. AMS I.49879‐001, 2 specimens, Saxon Reef, off Cairns, Queensland, Australia, 2022, preserved in RNAlater. Used for DNA extraction (GenBank PX508018, PX508019). Not examined morphologically (RNAlater preservation compromised bone integrity and body form).

### 4.1 Comparative material

*Solenostomus paegnius* Jordan & Thompson, 1914. All specimens were identified by the present authors.CAS 78331, 1 female, 54.3 mm SL, from New South Wales, Australia, died in transport to Steinhart Aquarium on 2 April 1977; originally identified as *Solenostomus* sp. This specimen was used for morphometrics and micro‐CT analysis.

CAS 240194, 1 male, 35.1 mm SL, Puerto Galera Bay, Coco Beach, Oriental Mindoro, Philippines, 13°31′ N, 120°57′ E, 11.4 m, 12 April 2015, coll. K. A. Miller, S. Cohen, S. Bedard, E. Ong & J. Comendador. Used for morphometrics, micro‐CT.

USNM 436291, 1 male, SL not measurable (caudal region damaged), Verde Island, San Agustin East, Batangas, Philippines, 13°33′ N, 121°04′ E, 12 m, 17 April 2015, collected by D. Catania & D. Dumale. Used for morphometrics and micro‐CT.

USNM 436453, 1 female, 39.5 mm SL, off the point between White Beach and Bayanan Beach, Puerto Galera, Oriental Mindoro, Philippines, 13°30′ N, 120°54′ E, 23.5 m, 30 April 2015, collected by D. Catania, D. Pitassy & D. Dumale. Used for morphometrics and micro‐CT.

SAIAB 39239, 1 female, 45.6 mm SL, East London Shooting Range, pool, Eastern Cape, South Africa, approximately 33° S, 28° E, 1–2 m, 13 April 1992; originally identified as *S. cyanopterus*. Used for morphometrics, micro‐CT.

## AUTHOR CONTRIBUTIONS

Graham Short and David Harasti conceived the study. Graham Short conducted morphological and molecular analyses. David Harasti provided field observations and photographic documentation. Both authors contributed to writing and editing the manuscript.

## Data Availability

The data that support the findings of this study are available from the corresponding author upon reasonable request. DNA sequences have been deposited in GenBank under accession numbers PX508018 and PX508019.
